# Comprehensive analysis of an immune infiltrate-related competitive endogenous RNA network reveals potential prognostic biomarkers for non-small cell lung cancer

**DOI:** 10.1371/journal.pone.0260720

**Published:** 2021-12-02

**Authors:** Cai-Zhi Yang, Lei-Hao Hu, Zhong-Yu Huang, Li Deng, Wei Guo, Shan Liu, Xi Xiao, Hong-Xing Yang, Jie-Tao Lin, Ling-Ling Sun, Li-Zhu Lin

**Affiliations:** 1 The First School of Medicine, Guangzhou University of Chinese Medicine, Guangzhou, China; 2 Integrated Chinese and Western Medicine Postdoctoral research station, Jinan University, Guangzhou, China; 3 Department of Radiotherapy, Guangdong Second Provincial General Hospital, Guangzhou, China; 4 Department of Oncology, the First Affiliated Hospital of Guangzhou University of Chinese Medicine, Guangzhou, China; 5 Postdoctoral Research Station, Guangzhou University of Chinese Medicine, Guangzhou, China; University of Science and Technology Liaoning, CHINA

## Abstract

Globally, non-small cell lung cancer (NSCLC) is the most common malignancy and its prognosis remains poor because of the lack of reliable early diagnostic biomarkers. The competitive endogenous RNA (ceRNA) network plays an important role in the tumorigenesis and prognosis of NSCLC. Tumor immune microenvironment (TIME) is valuable for predicting the response to immunotherapy and determining the prognosis of NSCLC patients. To understand the TIME-related ceRNA network, the RNA profiling datasets from the Genotype-Tissue Expression and The Cancer Genome Atlas databases were analyzed to identify the mRNAs, microRNAs, and lncRNAs associated with the differentially expressed genes. Weighted gene co-expression network analysis revealed that the brown module of mRNAs and the turquoise module of lncRNAs were the most important. Interactions among microRNAs, lncRNAs, and mRNAs were prognosticated using miRcode, miRDB, TargetScan, miRTarBase, and starBase databases. A prognostic model consisting of 13 mRNAs was established using univariate and multivariate Cox regression analyses and validated by the receiver operating characteristic (ROC) curve. The 22 immune infiltrating cell types were analyzed using the CIBERSORT algorithm, and results showed that the high-risk score of this model was related to poor prognosis and an immunosuppressive TIME. A lncRNA–miRNA–mRNA ceRNA network that included 69 differentially expressed lncRNAs (DElncRNAs) was constructed based on the five mRNAs obtained from the prognostic model. ROC survival analysis further showed that the seven DElncRNAs had a substantial prognostic value for the overall survival (OS) in NSCLC patients; the area under the curve was 0.65. In addition, the high-risk group showed drug resistance to several chemotherapeutic and targeted drugs including cisplatin, paclitaxel, docetaxel, gemcitabine, and gefitinib. The differential expression of five mRNAs and seven lncRNAs in the ceRNA network was supported by the results of the HPA database and RT-qPCR analyses. This comprehensive analysis of a ceRNA network identified a set of biomarkers for prognosis and TIME prediction in NSCLC.

## Introduction

Lung cancer is the leading cause of cancer-related deaths [1]. Non-small cell lung cancer (NSCLC) accounts for approximately 83% of all lung cancers [2]; its two dominant histological phenotypes include lung adenocarcinoma (LUAD, ~50%) and lung squamous cell carcinoma (LUSC, ~40%) [3]. Although remarkable advances have been made for lung cancer diagnoses and treatment strategies, 60-month overall survival (OS) rate and 5-year survival rates remain poor (68% and 0%–10% at stages IB and IV, respectively) [4]. Thus, accurate detection of NSCLC at an early stage can provide a good prognosis. However, localized diseases at stages I or II are diagnosed in only 16% of patients [5]. A low-dose computerized tomography scan is currently the most practical method for the early diagnosis of lung cancer [6]. However, its high false-positive rate of up to 96.4% [7] requires greater medical attention than needed and is an unnecessary psychological burden for the patients. Diagnostic precision can be enhanced by developing biomarkers that can accurately classify the patients according to their probable disease risk, diagnosis, and prognosis or response to treatment [8]. In addition, functional biomarkers with known underlying mechanisms related to the disease can be used as potential therapeutic targets [8]. For instance, identification of epidermal growth factor receptor (*EGFR*), anaplastic lymphoma kinase (*ALK*), c-ros proto-oncogene 1 receptor tyrosine kinase (*ROS1*), kirsten rat sarcoma viral oncogene homolog (*KRAS*), serine/threonine-protein kinase B-Raf *(BRAF*), mesenchymal-epithelial transition factor (*MET*), proto-oncogene tyrosine-protein kinase receptor Ret (*RET*), human epidermal growth factor receptor 2 (*HER2*), and neurotrophic receptor tyrosine kinase (*NTRK*) tyrosine kinase inhibitors (TKIs) have improved the outcomes for oncogene-predisposed NSCLC patients [9]. However, patient responses to TKIs are usually temporary because tumors eventually develop resistance to targeted therapies through on- or off-target mechanisms [10]. NSCLC patients show a positive response to immune checkpoint inhibitors (ICIs) that target programmed cell death-1 (PD-1)/programmed cell death ligand-1 (PD-L1) interaction [11]. Nevertheless, most NSCLC patients do not show any initial responses to ICIs, whereas others who respond for a certain period relapse due to an immunosuppressive microenvironment [12]. Thus, future studies should focus on possible options such as combination strategies involving an agent that can address a specific on- or off-target resistance mechanism upfront or during disease progression [12,13]. Therefore, the identification of novel molecular network biomarkers for early screen to improve prognosis and treatment in NSCLC is needed.

Cellular and molecular immune markers in the tumor immune microenvironment (TIME) play an important role in NSCLC development. The density and localization of tumor-infiltrating immune cells greatly affect the OS in NSCLC patients [14]. In NSCLC patients, a high intra-tumoral density of immature dendritic cells, regulatory T cells, and M2 macrophages has been associated with poor prognosis, whereas a high intra-tumoral density of CD8+ T cells, CD4+ T cells, M1 macrophages, mature dendritic cells, and natural killer cells has been correlated with better prognosis [14]. *PD-1* is the main immune checkpoint for immunotherapy in NSCLC. Several meta-analyses have recently shown a possible link between the *PD-1/PD-L1* axis and the prognosis of NSCLC patients [15]. The expression of chemokines, including *CXCL9*, *CXCL -10*, and *CXCL -16*, is also correlated with the prognosis of these patients [16]. Thus, the complex regulation of TIME in NSCLC must be examined to identify effective immune targets for accurate prognosis and increase the efficacy of clinical immunotherapy.

Pandolfi *et al*. [17] first proposed the competing endogenous RNA (ceRNA) hypothesis. Accordingly, a post-transcriptional regulatory network allows all RNA transcripts including mRNAs, long non-coding RNAs (lncRNAs), and circular RNAs, to regulate each other, by competing for shared microRNAs (miRNAs). miRNA response elements (MREs) are the keystone for the competitive binding of miRNAs with lncRNAs and mRNAs [18]. ceRNAs participate in the pathogenesis of multiple cancers, including NSCLC, and their abnormal expression is substantially associated with the prognosis of the patients [19,20]. Jiang *et al*. [21] constructed a ceRNA network to identify a prognostic signature in bladder cancer. Liu *et al*. [22] developed the seven-lncRNA prognostic signature in melanoma through an integrative analysis of the DElncRNA–DEmiRNA–DEmRNA ceRNA network. For the ceRNA network of NSCLC, through gene expression studies, some biomarkers associated with prognosis and tumor progression have been identified [23,24]. Based on the alternative splicing events, Li *et al*. [25] developed a high-performance prognostic predictor model for risk stratification in patients with NSCLC. The above studies have focused on the comparative analyses between limited peritumoral samples and paired tumor samples from The Cancer Genome Atlas (TCGA) database. However, this paradigm is far from ideal because of the differences in the transcriptomic profiles between para-tumor and healthy tissues [26]. The Genotype-Tissue Expression (GTEx) dataset is a large-scale public resource for genome-wide association studies in healthy human tissues [27]. GTEx and TCGA databases allow unprecedented studies of gene expression to address the limitation [28]. Weighted gene co-expression network analysis (WGCNA) is an effective approach to examine the intrinsic links between clinical outcomes and co-expression gene modules [29]; however, only a few studies have employed this method to analyze the relevance of co-expression patterns for ceRNAs.

In this study, a TIME-associated ceRNA regulatory network for the prognostic prediction of NSCLC was constructed using the data from TCGA and GTEx databases by multiple bioinformatic analytic methods including WGCNA and The Cell Type Identification by Estimating Relative Subsets of RNA Transcripts (CIBERSORT).

## Results

### Analysis of differentially expressed genes between TCGA and GTEx samples

**Fig 1:**
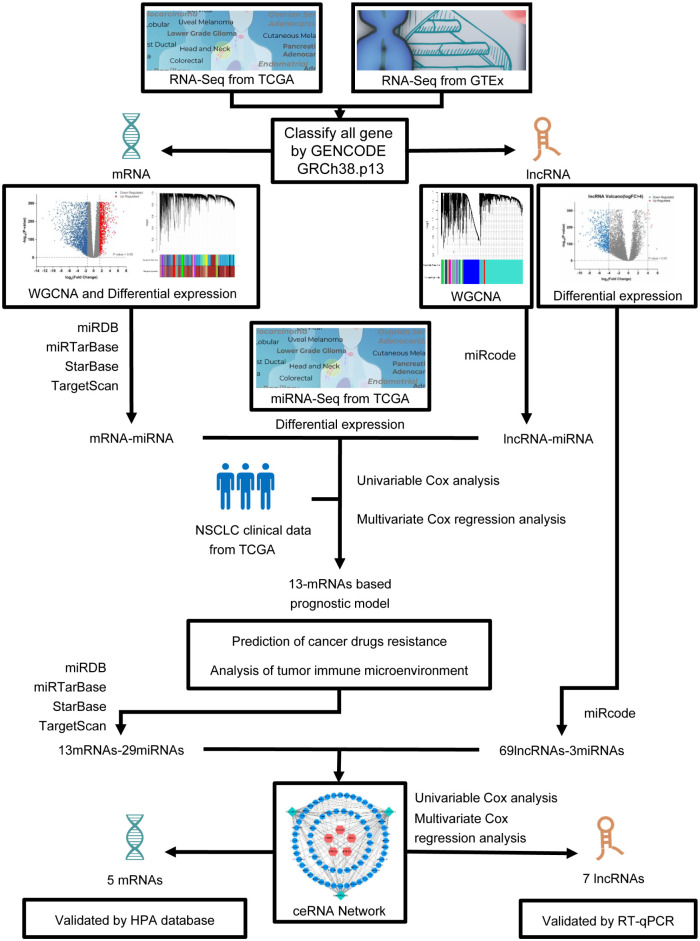
Flow diagram of study process.

**Fig 2:**
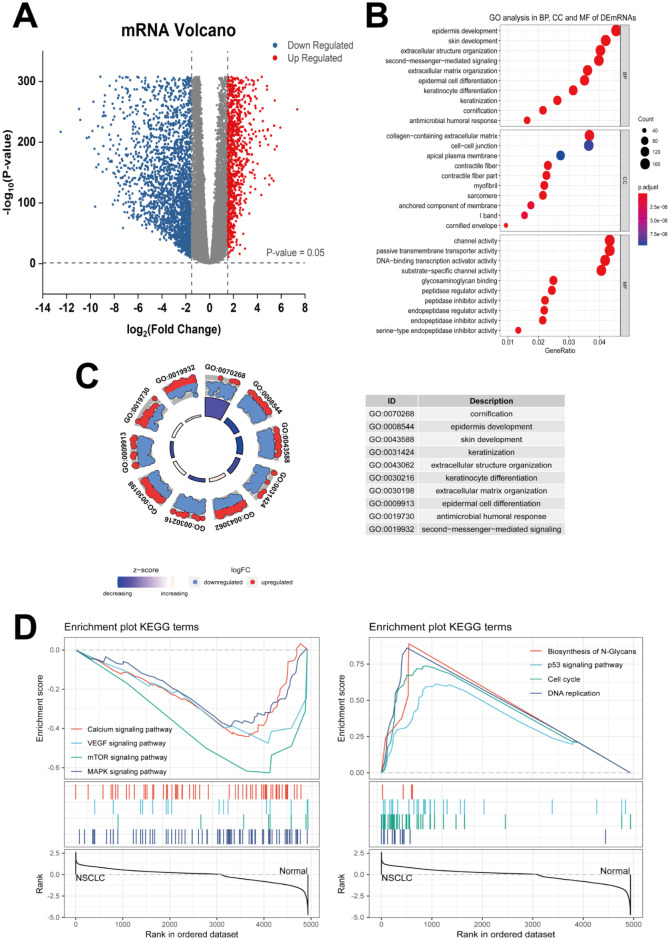
Analysis of DEmRNAs from TCGA and GTEx. (**A**) Volcano map of important DEmRNAs including 1838 up-regulated and 3097 down-regulated mRNAs. (**B**) GO analysis showing the involvement of BP, CC, and MF in DEmRNAs. (**C**) Distribution of DEmRNAs in BP. Red circles display up-regulated genes, and blue ones show down-regulated genes in the scatter plot of outer circle. (**D**) KEGG-GSEA was applied to the signaling pathway enrichment of DEmRNAs. TCGA, the Cancer Genome Atlas database; GTEx, the Genotype–Tissue Expression database; DEmRNAs, differentially expressed mRNAs; BP, biological process; CC, cellular component; MF, molecular function; GO, Gene Ontology.

Fig 1 depicts the study design. The RNA expression levels in 944 NSCLC samples and 578 normal lung samples were evaluated. A total of 1838 significantly up-regulated mRNAs and 3097 down-regulated mRNAs were identified and termed as differentially expressed mRNAs (DEmRNAs) in NSCLC; their distribution is shown in Fig 2A. Gene Ontology (GO) analysis was performed to identify the enriched biological mechanisms underlying DEmRNAs, and the GO enrichment results are shown in Fig 2B. In the biological process (BP), DEmRNAs were significantly enriched in cornification, epidermis development, skin development, keratinization, extracellular structure organization, and keratinocyte differentiation. The Z-score of GO enrichment was closer to blue module, which indicated that most of the BPs were likely under-represented (Fig 2C). Gene Set Enrichment Analysis (GSEA) showed that the N-glycan biosynthesis, *p53* signaling pathway, cell cycle, and DNA replication-related genes were up-regulated, whereas calcium, VEGF, mTOR, and MAPK signaling pathways were significantly down-regulated (Fig 2D).

### Analyses of mRNA modules by WGCNA

**Fig 3:**
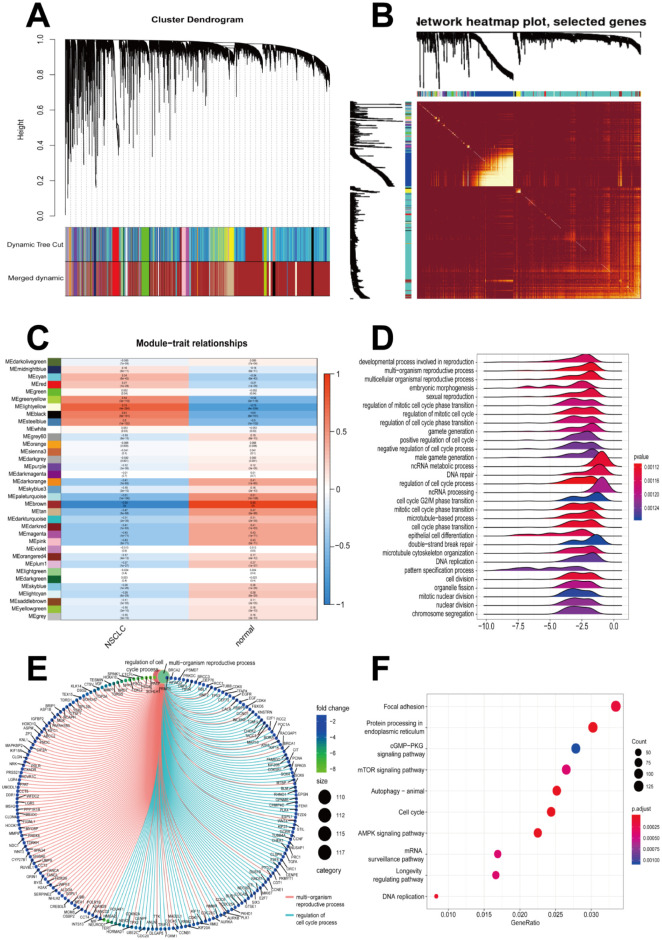
Analysis of mRNA modules by WGCNA. **(A)** Dendrogram of mRNAs with top 80% variances clustered based on topological overlap. **(B)** TOM heatmap plot of selected genes. Light colors denote low topological overlap, and dark colors denote high topological overlap. **(C)** Correlation of gene color modules between NSCLC and normal samples. The ME of the brown module was the most positively related to NSCLC. **(D–E)** GO-GSEA showing the gene symbols and gene interaction in the brown module. **(F)** KEGG investigating the signal pathway enrichment in the brown module. WGCNA, weighted gene co-expression network analysis; TOM, topological overlap matrix; NSCLC, non-small cell lung cancer; ME, module eigengene; GO, Gene Ontology; KEGG, Kyoto Encyclopedia of Genes and Genomes pathway analyses; GSEA, Gene Set Enrichment Analysis.

The top 80% mRNAs (14428 genes) obtained from variance comparison were used to construct the gene modules using the WGCNA algorithm. Soft-thresholding was set at β = 8 and module size cut-off at 25 to characterize a scale-free network. After the co-expressed genes were categorized by average linkage clustering, a total of 35 gene color modules were identified (Fig 3A). Unassigned genes were categorized into the gray module. A total of 1000 genes from the mRNA color modules were randomly chosen to plot the network heatmap and visualize the topological overlap matrix (TOM) (Fig 3B). Among the 35 color modules, the module eigengene (ME) of the brown module showed the highest association with NSCLC and normal traits (Fig 3C). Thus, the brown module containing 9041 genes was considered as the key module for NSCLC. GO term enrichment analysis was performed for the mRNAs in the brown module and the functional enrichment and gene interactions in BPs were identified (Fig 3D and 3E). These genes were mainly related to the developmental processes in reproduction, multi-organism reproductive processes, and cell cycle regulation; these were clustered in focal adhesion, protein processing in the endoplasmic reticulum, mTOR signaling pathway, and autophagy according to the Kyoto Encyclopedia of Genes and Genomes (KEGG) pathway enrichment analysis (Fig 3F).

### Analysis of lncRNA modules by WGCNA and prediction of target genes

**Fig 4:**
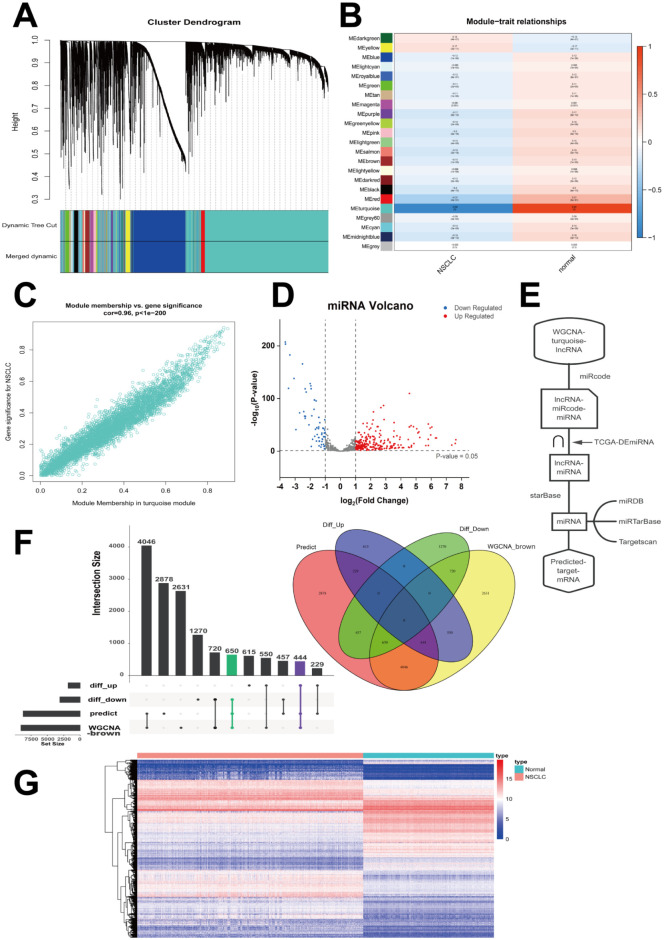
Analysis of lncRNA modules by WGCNA and prediction of target genes. **(A)** Cluster dendrogram of lncRNAs with top 60% variances clustered based on topological overlap. **(B)** Correlation of lncRNA modules between NSCLC and normal samples. The ME of the turquoise module was the most positively related to traits. **(C)** Relationship between gene significance and module membership in the turquoise module. **(D)** Volcano map of 316 DEmiRNAs (|logFC| > 1, *P* < 0.05) in NSCLC. **(E)** Flow chart of target mRNA prediction. **(F)** Overlapped target mRNAs were analyzed by the predicted target mRNAs, WGCNA-brown mRNAs, and the significantly up- or down-regulated mRNAs. **(G)** Expression heatmap displaying the 1094 predicted target mRNAs in NSCLC. WGCNA, weighted gene co-expression network analysis; NSCLC, non-small cell lung cancer; ME, module eigengene.

The constructed lncRNA network was used to identify the hub modules. The top 60% lncRNAs (8102 genes) obtained from variance comparison were analyzed by WGCNA. Soft-thresholding was set at β = 3 and module size cut-off at 25. A total of 23 co-expressed lncRNA modules were identified (Fig 4A). Among them, the turquoise module, which included 1587 lncRNAs as shown in Fig 4B, was the most significantly associated with NSCLC (r = 0.92). Furthermore, lncRNAs in the turquoise module had the most remarkable correlation with gene significance for NSCLC in all modules (Fig 4C, cor = 0.96). A total of 316 differentially expressed miRNAs (DEmiRNAs) were obtained from the comparison of TCGA miRNA-seq data between 961 NSCLC and 90 normal lung tissue samples (Fig 4D). The lncRNA–miRcode–miRNA relationship between 1587 differentially expressed lncRNAs (DElncRNAs) in the turquoise module and 316 DEmiRNAs was evaluated using the online miRcode tool. Overlapping miRNAs (60) between lncRNA–miRcode–miRNA (326) and DEmiRNAs (316) were then obtained. Using miRNA target prediction tools, 60 common miRNAs from the intersection were used for subsequent analyses. A total of 8704 predicted target mRNAs were acquired (Fig 4E). As shown in Fig 4F, 1094 mRNAs were obtained through the interactions of the 8704 predicted mRNAs and 4935 DEmRNAs (1838 up-regulated and 3097 down-regulated mRNAs) with 9041 mRNAs in the brown module. The 1094 genes in 1577 samples are shown using an expression heatmap in Fig 4G.

### Construction of 13-mRNA-based prognostic model to predict chemotherapeutic response

**Fig 5:**
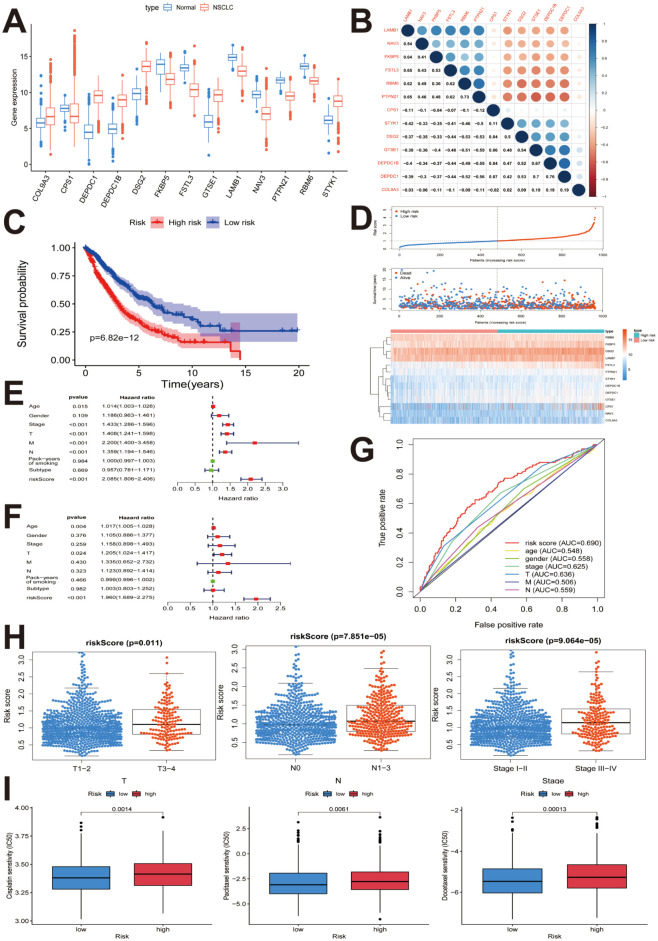
Construction of the 13-mRNA-based prognostic model to predict chemotherapeutic response. **(A)** Differential expression of 13 selected genes between NSCLC and normal samples. **(B)** Relationship of each gene in the 13 gene-based model. **(C)** Risk score distribution in patients with NSCLC, survival status in different groups, and heatmap of the expression of 13 genes. **(D)** K–M plot represents that patients in the high-risk group had significantly shorter OS than those in the low-risk group. **(E)** Forest plot of univariate Cox regression analysis showing the association between risk factors and NSCLC survival. **(F)** Forest plot of multivariate Cox regression analysis showing that the 13-mRNA signature was an independent predictor of prognosis in NSCLC. **(G)** ROC curve showed that AUC of 13-mRNA prognostic model was 0.690. **(H)** Clinicopathological significance of 13 gene-related prognostic model in NSCLC. **(I)** Relation of the risk score to the estimated IC50 of cisplatin, paclitaxel, and docetaxel. NSCLC, non-small cell lung cancer; K-M, Kaplan–Meier survival analysis; ROC, receiver operating characteristic curve analysis; AUC, the area under the curve; IC50, half maximal inhibitory concentration.

TCGA patient data including OS and clinical features were obtained for 944 NSCLC patients. The clinicopathological characteristics of NSCLC patients are shown in Table 1. A univariate regression analysis was performed to investigate the relevance of the expression levels of the 1094 target genes with OS in NSCLC. The cut-off for significant association was set at *p* < 0.05. As a result, 31 genes were obtained and further included in the subsequent multivariate cox regression analysis (Table 2). A prognostic model for OS was constructed using the following 13 genes: *FSTL3*, *CPS1*, *PTPN21*, *DEPDC1B*, *COL9A3*, *DSG2*, *LAMB1*, *STYK1*, *RBM6*, *DEPDC1*, *GTSE1*, *NAV3*, and *FKBP5*. As shown in Fig 5A, D*EPDC1*, *COL9A3*, *STYK1*, *DSG2*, *GTSE1*, and *DEPDC1B* were up-regulated, whereas *RBM6*, *LAMB1*, *CPS1*, *NAV3*, *FKBP5*, *FSTL3*, and *PTPN21* were down-regulated in patients with NSCLC. Fig 5B displays the correlation between the expressions of each gene in the 13 gene-based model. The risk assessment scoring was computed for each NSCLC patient, and the threshold for risk score was set at 1.006. Accordingly, the patients were divided into high- (n = 471) and low-risk (n = 473) groups as shown in Table 3. The accuracy of the 13 gene-based prognostic model in predicting NSCLC clinical outcomes were estimated by Kaplan–Meier (K–M) survival analysis, Cox proportional hazard regression model, and ROC. As shown in Fig 5C, the K–M survival curves showed that OS in the high-risk group was significantly shorter (*P* < 0.001) than that for patients with low-risk prediction. Fig 5D shows the comparison between the risk score distribution, survival status, and expression heatmap for 13 genes between the high- and low-risk groups. According to the univariate analysis, OS was significantly associated with the risk score and the TNM, T, N, and M stages (*P*<0.001) (Fig 5E). However, multivariate analysis indicated that among the above-mentioned prognostic factors, the risk score was the only independent prognostic predictor (HR = 1.960, 95% CI = 1.689–2.275, *P*<0.001) (Fig 5F). The area under the curve (AUC) for the risk score was 0.690 (Fig 5G); higher than that for other clinical factors. The relationship between the clinicopathological parameters and the 13 gene-based prognostic model is shown in Fig 5H. The values for the prognostic prediction model were higher in T3-4 than in T1-2 stages (*P* = 0.011); in N1-3 than in N0 (*P* < 0.001), and in stages III–IV than in stages I–II (*P* < 0.001). The estimated half-maximal inhibitory concentrations (IC50) of three commonly used NSCLC chemotherapeutic agents, including, cisplatin, docetaxel, and paclitaxel, were compared between the low- and high-risk groups of patients using the pRRophetic algorithm. The three drugs showed higher IC50 values and lower sensitivity in the high-risk as compared to low-risk groups (all *P* < 0.01) (Fig 5I). Thus, the result suggested that high-risk patients with NSCLC exhibit resistance to cisplatin, docetaxel, and paclitaxel. Taken together, the prognostic model was effective in predicting the therapeutic response of high- and low-risk NSCLC patients towards the three drugs.

**Table 1:** The clinicopathological characteristics of 944 NSCLC patients.

Parameters	LUSC (n = 489)	LUAD (n = 455)	Total(n = 944)
**Gender**			
FEMALE	127 (25.97%)	248 (54.51%)	375 (39.7%)
MALE	362 (74.03%)	207 (45.49%)	569 (60.3%)
**Smoking history**			
No	75 (15.34%)	142 (31.21%)	217 (23.0%)
Yes	414 (84.66%)	313 (68.79%)	727 (77.0%)
**Age**			
Mean(SD)	66.4847 (9.3926)	65.778 (9.318)	66.1441 (9.3584)
Median[Min, Max]	67 [33, 88]	67 [38, 90]	67 [33, 90]
**Survival status**			
Dead	278 (56.85%)	282 (61.98%)	560 (59.3%)
Alive	211 (43.15%)	173 (38.02%)	384 (40.7%)
**TNM Stage**			
Stage I	239 (48.88%)	246 (54.07%)	485 (51.4%)
Stage II	158 (32.31%)	110 (24.18%)	268 (28.4%)
Stage III	85 (17.38%)	75 (16.48%)	160 (16.9%)
Stage IV	7 (1.43%)	24 (5.27%)	31 (3.3%)

**Table 2:** Univariate and multivariate cox proportional hazard regression analysis of 31 genes.

Gene	Univariate		Multivariate	
	HR(95%CI)	*P*	HR(95%CI)	*P*
*FSTL3*	1.195(1.109–1.288)	<0.0001	1.178(1.078–1.287)	0.000294 **
*ANLN*	1.156(1.067–1.252)	0.0004		
*HMMR*	1.162(1.060–1.273)	0.0014		
*PRR11*	1.154(1.057–1.260)	0.0014		
*ARNTL2*	1.099(1.034–1.169)	0.0024		
*DSG2*	1.158(1.053–1.273)	0.0025	1.118(0.993–1.259)	0.065513
*CPS1*	1.048(1.016–1.080)	0.0028	1.053(1.018–1.089)	0.002604 **
*DEPDC1B*	1.119(1.035–1.209)	0.0046	1.226(1.046–1.437)	0.012009 *
*PKM*	1.219(1.062–1.399)	0.0049		
*DCBLD2*	1.131(1.037–1.232)	0.0053		
*NAV3*	1.104(1.028–1.186)	0.0067	1.066(0.981–1.158)	0.130179
*KIF4A*	1.115(1.029–1.207)	0.0076		
*AURKA*	1.137(1.034–1.250)	0.0080		
*GTSE1*	1.112(1.026–1.205)	0.0096	1.154(0.979–1.361)	0.087804
*NGEF*	1.081(1.018–1.149)	0.0117		
*PTPN21*	1.150(1.030–1.283)	0.0129	1.203(1.044–1.386)	0.010568 *
*FKBP5*	1.115(1.022–1.218)	0.0149	1.075(0.977–1.183)	0.137657
*CLSPN*	1.104(1.018–1.198)	0.0164		
*DEPDC1*	1.094(1.016–1.178)	0.0167	0.864(0.733–1.019)	0.083033
*LAMB1*	1.118(1.017–1.230)	0.0211	0.890(0.785–1.009)	0.068225
*STYK1*	1.109(1.015–1.212)	0.0218	1.092(0.989–1.206)	0.081164
*NDC80*	1.095(1.010–1.187)	0.0271		
*ITIH4*	0.922(0.855–0.993)	0.0320		
*RBM6*	0.856(0.741–0.989)	0.0346	0.860(0.726–1.019)	0.082
*CDC6*	1.087(1.005–1.175)	0.0364		
*ADAMTS6*	1.095(1.005–1.193)	0.0376		
*COL9A3*	1.055(1.002–1.111)	0.0401	1.067(1.009–1.129)	0.024166 *
*AASS*	0.917(0.845–0.996)	0.0408		
*MCM10*	1.077(1.002–1.158)	0.0436		
*NEXMIF*	0.956(0.915–0.999)	0.0455		
*SCIN*	0.952(0.907–1.000)	0.0489		

**Table 3:** The association between risk score of the 13-gene-based prognostic model and clinicopathological characteristics.

Parameters	Low risk (n = 473)	High risk (n = 471)	χ^2^	*P* Value
**Age**				
≤ 65	215 (52.31%)	196 (47.69%)	1.4163	0.234
> 65	258 (48.41%)	275 (51.59%)		
**Gender**				
FEMALE	210 (56.00%)	165 (44.00%)	8.6454	0.0033 **
MALE	263 (46.22%)	306 (53.78%)		
**Subtype**				
LUSC	224 (45.81%)	265 (54.19%)	7.4972	0.0062 **
LUAD	249 (54.73%)	206 (45.27%)		
**Smoking history**				
No	119 (54.84%)	98 (45.16%)	2.5246	0.1121
Yes	354 (48.69%)	373 (51.31%)		
**T stage**				
T1-2	406 (51.20%)	387 (48.80%)	2.3649	0.1241
T3-4	67 (44.37%)	84 (55.63%)		
**N stage**				
N0	333 (53.54%)	289 (46.46%)	8.5866	0.0034 **
N1-3	140 (43.48%)	182 (56.52%)		
**TNM stage**				
Stage I-II	393 (52.19%)	360 (47.81%)	6.4734	0.0109 *
Stage III-IV	80 (41.88%)	111 (58.12%)		

### Construction of a ceRNA network

**Fig 6:**
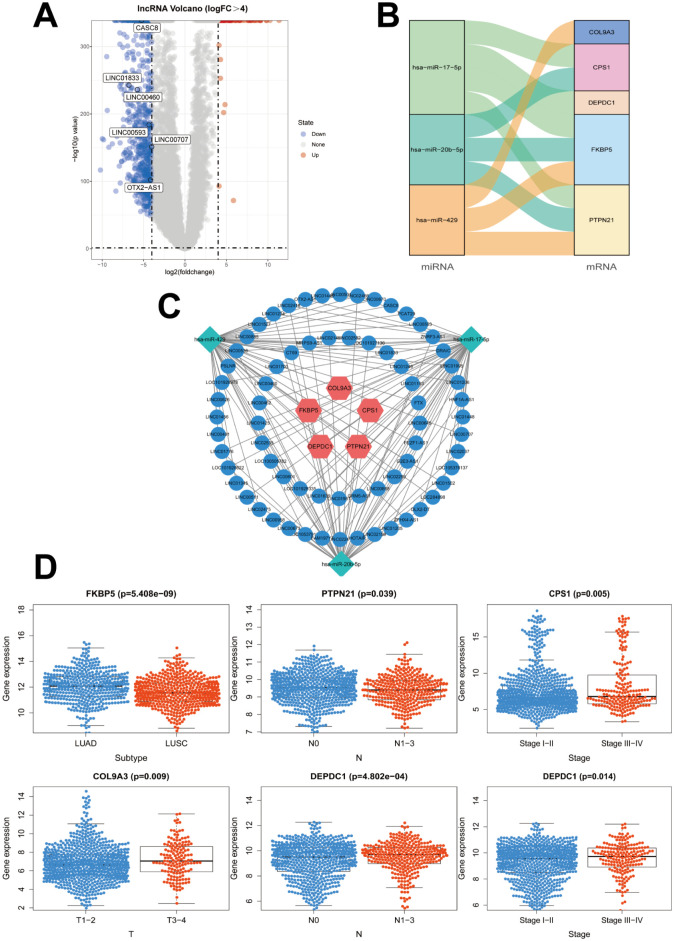
Construction of a lncRNA–miRNA–mRNA ceRNA network. **(A)** Volcano map of 69 significant DElncRNAs. **(B)** Correlation between the five prognostic mRNAs and their corresponding miRNAs. **(C)** A ceRNA network was constructed containing five mRNAs, three miRNAs, and 69 lncRNAs for NSCLC prognosis. **(D)** Clinicopathological significance of *FKBP5*, *DEPDC1*, *CPS1*, *COL9A3*, and *PTPN21* in NSCLC. ceRNA, Competing endogenous RNA; DElncRNAs, differentially expressed lncRNAs; NSCLC, non-small cell lung cancer.

Fig 1 shows the established lncRNA–miRNA–mRNA ceRNA network obtained through the prognostic mRNA signatures, predicted miRNAs, and DElncRNAs. First, 29 miRNAs were obtained through prediction using the 13 mRNAs. After the analysis of lncRNA expression data from TCGA database, 69 DElncRNAs [|log (fold change)| (|logFC|) > 4, *P* < 0.05] were identified through the empirical analysis of digital gene expression in R (edgeR) (Fig 6A). However, only three miRNAs were further selected. These three miRNAs were retained after overlapping with the 29 predicted miRNAs. Thus, five mRNAs were obtained from the 13 prognostic mRNAs which corresponded to the three miRNAs (Fig 6B). Finally, a ceRNA network containing five mRNAs, three miRNAs, and 69 lncRNAs was constructed as shown in Fig 6C. The relationships between clinicopathological parameters and expression of the five mRNAs in the ceRNA network are shown in Fig 6D.

### Relationship between the risk score from ceRNA network and tumor immune microenvironment

**Fig 7:**
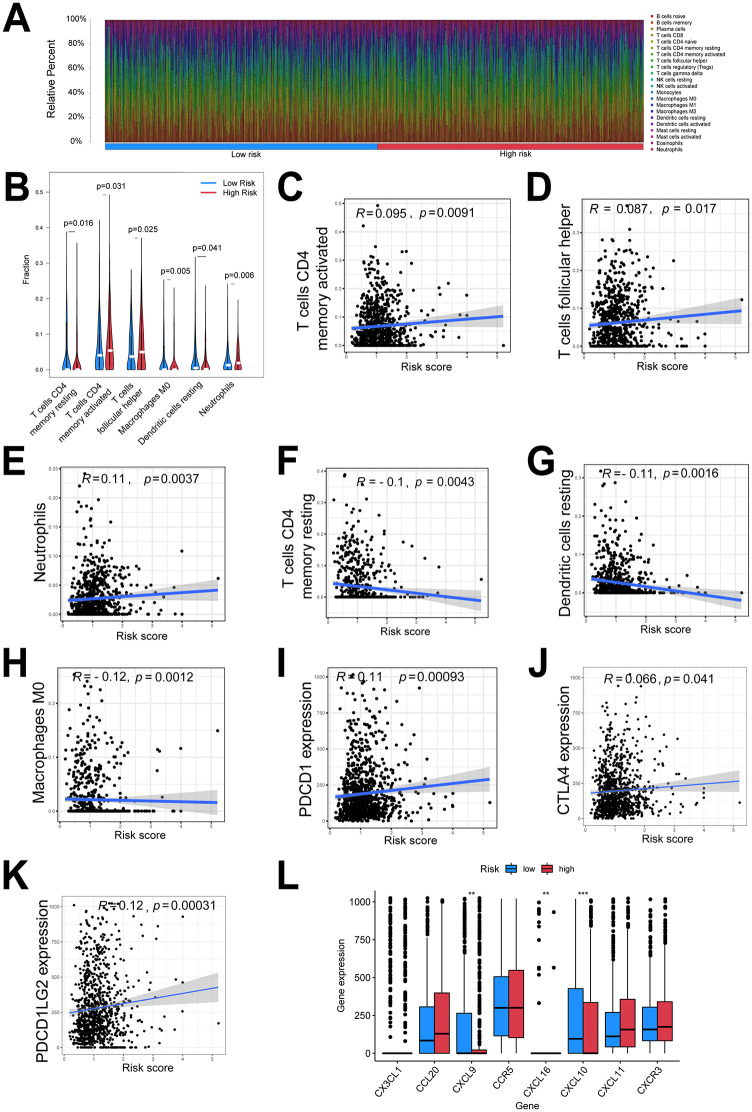
Relationships between the risk score of ceRNA network and tumor immune microenvironment. **(A)** Landscape of immune cell infiltration between low- and high-risk patients with NSCLC. **(B)** Fractions of significantly differential immune cell types between low- and high-risk patients with NSCLC. **(C–H)** Correlations between risk score and infiltration levels of significantly different immune cell types. **(I)** Comparison of PD-1 expression level between low- and high-risk patients with NSCLC. **(J)** Correlations between risk score and PD-1 expression levels. **(K)** Comparison of expression level of chemokines between low- and high-risk patients with NSCLC. NSCLC, non-small cell lung cancer; PD-1, programmed cell death protein 1.

The differences in the 22 infiltrating immune cell types were determined between low- and high-risk NSCLC patients using the CIBERSORT algorithm. Fig 7A summarizes the proportion of immune cell types in 944 patients from the TCGA database. The high-risk patients had higher infiltration levels of activated CD4 memory and follicular helper T cells (Fig 7B and 7D), whereas greater infiltration of CD4 memory resting T cells, M0 macrophages, resting dendritic cells, and neutrophils were observed in low-risk patients (Fig 7B and 7E–7H). Given the important role of chemokines and immune checkpoints in TIME and immune response, we analyzed the correlation between the expression of these molecules and the risk score. The expression of PD-1, positively associated with the risk score, was significantly upregulated in the high-risk group as compared to the low-risk group (Fig 7I and 7J). In addition, the expression of chemokines for immune activation (i.e., CXCL9, CXCL10, and CXCL16) was significantly lower in the high-risk group than in the low-risk group (Fig 7K). These results indicated that ceRNA risk signatures may be implicated in the NSCLC immunosuppressive microenvironment.

### Construction of seven-lncRNA-based prognostic model from ceRNA network to predict drug resistance in cancer

**Fig 8:**
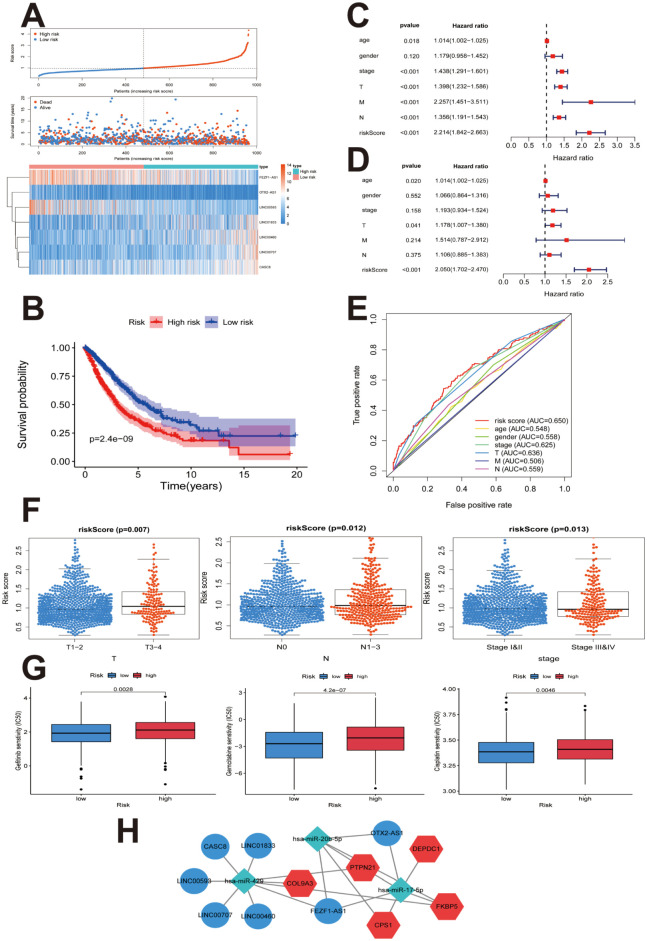
Construction of the seven-lncRNA-based prognostic model from ceRNA network to predict resistance to cancer drugs. **(A)** Risk score distribution in patients with NSCLC, survival status in different groups, and heatmap of the expression of seven lncRNAs. **(B)** K–M plot represents that patients in the high-risk group had significantly shorter OS than those in the low-risk group. **(C)** Forest plot of univariate Cox regression analysis showed the association between risk factors and survival of NSCLC. **(D)** Forest plot of multivariate Cox regression analysis showing that the seven-lncRNA signature was an independent predictor of prognosis in NSCLC. **(E)** ROC curve showing that the AUC of seven-lncRNA prognostic model was 0.650. **(F)** Clinicopathological significance of seven-lncRNA-related prognostic model in NSCLC. **(G)** Relation between the lncRNA risk score and the estimated IC50 of gefitinib, gemcitabine, and cisplatin. **(H)** Hub ceRNAs from coregulatory network involving five mRNAs from the 13gene-based prognostic model and three miRNAs from the seven-lncRNA signature. ceRNA, Competing endogenous RNA; NSCLC, non-small cell lung cancer; K-M, Kaplan–Meier survival analysis; ROC, receiver operating characteristic curve analysis; AUC, the area under the curve.

The results of lncRNA analysis were similar to the findings through mRNA analysis. Univariate regression analysis was performed to evaluate the relevance of the expression of the 69 lncRNAs from the ceRNA network with OS. Nine lncRNAs were obtained by setting the cut-off for significant association at *P* < 0.05. These were further included for the subsequent multivariate cox regression analysis (Table 4). A prognosis model for OS was constructed with the following seven lncRNAs: *LINC00707*, *LINC00460*, *FEZF1-AS1*, *LINC00593*, *OTX2-AS1*, *LINC01833*, and *CASC8*. The risk score was calculated for each NSCLC patient, and the threshold was set at 0.9755. Accordingly, the patients were classified into high- (n = 471) and low-risk (n = 473) groups. Fig 8A shows the distribution of risk scores for NSCLC patients, the survival status in different groups, and the heatmap of the expression of seven lncRNAs. The accuracy of the seven-lncRNA-based prognostic model in predicting NSCLC clinical outcomes was estimated by K–M survival analysis, Cox regression model, and ROC. As shown in Fig 8B, the K–M survival curves showed that OS in the high-risk group was significantly shorter than that in patients with low predicted risk (*P* < 0.001). According to the univariate analysis, the risk score and TNM, T, N, and M stages were significantly correlated with OS in NSCLC (*P*<0.001) (Fig 8C). However, multivariate analysis results showed that among them, only the risk score was an independent prognostic predictor (HR = 2.050, 95% CI = 1.702–2.470, *P*<0.001) (Fig 8D). The AUC for risk score was 0.650 (Fig 8E); higher than those for other clinical factors. Fig 8F illustrates the relationship between the clinicopathological parameters and the seven-lncRNA-based model. Higher prognostic prediction values were found in T3-4 than in T1-2 (*P* = 0.007) stages; in N1-3 than in N0 (*P* = 0.012), and in stages III–IV than in stages I–II (*P* = 0.013). Gefitinib, gemcitabine, and cisplatin are drugs commonly used in NSCLC treatment [30]. The estimated IC50 values of these drugs for the low- and high-risk patients were compared using the pRRophetic algorithm. All three drugs had higher IC50 values and lower sensitivity in the high-risk patients as compared to those for low-risk patients (all *P* < 0.01) (Fig 8G). This result indicated that high-risk NSCLC patients were resistant to gefitinib, gemcitabine, and cisplatin. Based on the seven-lncRNA signature, a prognostic ceRNA network involving five mRNAs of the 13 gene-based prognostic model and three miRNAs were extracted (Fig 8H).

**Table 4:** Univariate and multivariate cox proportional hazard regression analysis of 9 lncRNAs.

lncRNA	Univariate		Multivariate	
	HR(95%CI)	*P*	HR(95%CI)	*P*
*LINC00707*	1.092(1.045–1.141)	0.0001	1.051(1.004–1.099)	0.0316 *
*LINC00460*	1.073(1.033–1.115)	0.0003	1.044(1.004–1.085)	0.0314 *
*FEZF1-AS1*	0.944(0.911–0.980)	0.0021	0.964(0.927–1.002)	0.0650
*LINC00593*	0.937(0.898–0.977)	0.0024	0.943(0.902–0.986)	0.0094 **
*OTX2-AS1*	0.907(0.850–0.968)	0.0034	0.902(0.842–0.968)	0.0039 **
*LINC02535*	1.079(1.025–1.136)	0.0039		
*LINC01833*	1.050(1.015–1.087)	0.0049	1.055(1.020–1.092)	0.0021 **
*CASC8*	1.061(1.014–1.110)	0.0097	1.054(1.004–1.106)	0.0331 *
*LINC00601*	1.059(1.001–1.120)	0.0465		

### Validation of the risk mRNAs and risk lncRNAs in ceRNA network

**Fig 9:**
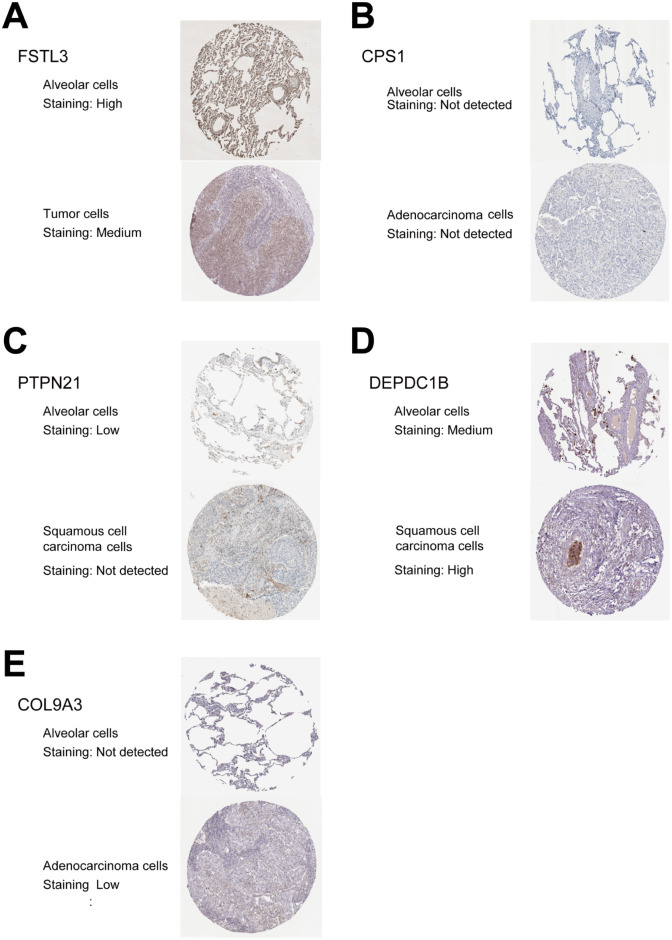
Validation of the risk mRNAs in ceRNA network by using the human protein atlas database. **(A-E)** Validation of FSTL3, CPS1, PTPN21, DEPDC1B, and COL9A3 by The Human Protein Atlas database (IHC) at the protein level. ceRNA, Competing endogenous RNA; IHC, immunohistochemistry.

We analyzed the immunohistochemical staining data obtained from the Human Protein Atlas (HPA) database to compare the expression levels of five risk genes (*FSTL3*, *CPS1*, *PTPN21*, *DEPDC1B*, and *COL9A3*) from the seven-lncRNA-based ceRNA network. The results showed that the expression at the protein level for two risk genes (*DEPDC1B* and *COL9A3*) was significantly higher in LUSC or LUAD tissues than in normal lung tissues (Fig 9D and 9E); two genes (*FSTL3* and *PTPN21*) showed an opposing trend (Fig 9A and 9C), which was also consistent at the transcriptional level (Fig 5A). Only *CPS1* expression at the protein level was not detected in both the normal lung and NSCLC tissues in the HPA database (Fig 9B).

**Fig 10:**
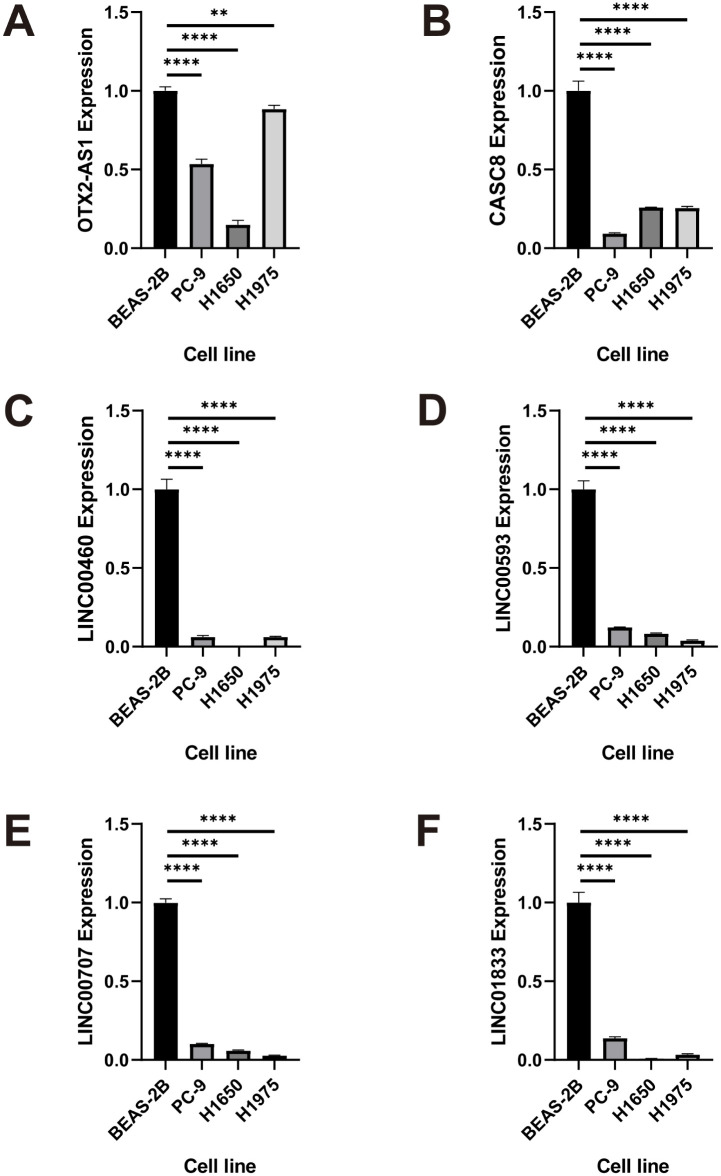
Validation of lncRNAs by RT-qPCR. **(A-F)** Relative expression levels of *LINC00707*, *LINC00460*, *LINC00593*, *LINC01833*, and *CASC8* in normal and NSCLC cell lines. Experiments were performed in triplicate, * *P* < 0.05, ** *P* < 0.01, *** *P* < 0.001, **** *P* < 0.0001 by ANOVA. RT-qPCR, quantitative reverse transcription polymerase chain reaction; NSCLC, non-small cell lung cancer.

In the GTEx and TCGA databases, the gene expression levels of six OS-related lncRNAs (*LINC00707*, *LINC00460*, *FEZF1-AS1*, *LINC00593*, *OTX2-AS1*, *LINC01833*, and *CASC8*) were significantly lower in NSCLC as compared to the normal lung tissues (Fig 6A). The results of reverse transcription-quantitative polymerase chain reaction (RT-qPCR) showed that the gene expression levels of *LINC00707*, *LINC00460*, *OTX2-AS1*, *LINC01833*, *LINC00593*, and *CASC8* were significantly lower in PC-9, H1650, and H1975 cells as compared to the BEAS-2B cell line (Fig 10A–10F).

## Discussion

NSCLC is one of the leading causes of cancer-related death, worldwide [31]. Despite the rapid development in immunotherapy and targeted therapy for NSCLC, the 5-year overall survival of NSCLC patients remains low, due to the lack of availability of effective biomarkers for early diagnosis and a poor understanding of the pathogenesis of NSCLC. Powerful computational models to predict potential disease-related non-coding RNAs for experimental validation are helpful for in-depth interpretation of the pathogenesis and processes underlying NSCLC development and improving related treatment strategies, and may dramatically decrease the time and expenditure on biological experiments [32–35]. The ceRNA network hypothesis was proposed to describe a new RNA regulatory crosstalk where different RNA transcripts can potentially regulate each other through MREs [17]. This concept unfolds a novel paradigm for the lncRNA–miRNA–mRNA regulatory network that may explain the mechanism underlying tumorigenesis. Recently, the advancement in interaction prediction studies using computational biology provides valuable insights into the development of lncRNA-mediated ceRNA network [36–38]. TIME composition is strongly associated with neoplastic progression, anti-tumor immune response, and clinical outcome in NSCLC [14,39]. Therefore, an immune-related ceRNA network must be explored to improve the accuracy of prognostic prediction and clinical response to immunotherapy. To the best of our knowledge, this is the first study that establishes an immune-related ceRNA prognostic model based on GTEx-TCGA data, WGCNA, pRRophetic algorithm, and TIME in NSCLC.

In this study, the RNA-Seq data from 944 NSCLC and 578 normal lung specimens were acquired from TCGA and GTEx databases, respectively. Key mRNAs and lncRNA signatures associated with NSCLC prognosis were identified using bioinformatic methods, including edgeR, WGCNA, GO term enrichment analysis, GSEA, KEGG pathway enrichment analysis, ceRNA network construction, and Cox regression analyses. A ceRNA network containing five mRNA signatures, seven lncRNA signatures, and three miRNAs were significantly related to OS in NSCLC patients. Additionally, correlations between the risk score of ceRNA prognostic model and TIME, and with sensitivity towards cancer drugs were investigated. Finally, immunohistochemical data from the HPA database and RT-qPCR were used to validate the findings from this study.

The lncRNA–miRNA–mRNA ceRNA network has an important influence on NSCLC pathogenesis and prognosis. Wang *et al*. [23] investigated a ceRNA network including 113 DElncRNAs, 12 DEmiRNAs, and 36 DEmRNAs with significant association with NSCLC. Wang *et al*. [40] constructed a ceRNA network involving 155 lncRNAs, 30 miRNAs, and 68 mRNAs to identify novel targets and pathways in NSCLC. In both these studies, the tumor and non-tumor samples were obtained from the same patients from the TCGA database to accurately elucidate the ceRNA interactive mechanism underlying NSCLC biology. Their findings highlight the prognostic value of ceRNA for NSCLC patients. However, the number of normal samples in the TCGA-NSCLC cohort is limited. Biomolecules secreted by cancer cells can be engulfed by the neighboring normal cells, thereby altering the gene expression and biological processes in these two cells [41]. The idea that cancer-related genetic aberrancy also exists in para cancer tissues adjacent to the highly genetically abnormal tumor tissues may be biased [42]. Hence, the use of a large number of normal samples from individuals with no tumors is necessary [43]. In the present work, edgeR was used to identify the DEmRNAs between NSCLC and normal lung samples. KEGG–GSEA results showed that DEmRNAs were enriched in the cell cycle, DNA replication-related genes, and the *mTOR* pathway. Further WGCNA analysis showed that the brown gene modules were significantly related to NSCLC. GO term enrichment results showed that the mRNAs in the brown modules were involved in cell cycle regulation, and KEGG analysis showed that they were clustered in the *mTOR* signaling pathway and DNA replication processes. *PI3K/Akt/mTOR* signaling commonly occurs in tumors and controls DNA replication and replisome stability, thereby regulating cell cycle progression [44]. Therefore, *PI3K/Akt/mTOR* signaling may be an essential mechanism underlying NSCLC initiation and progression.

Only a few ceRNA network studies discuss the relationship of expression patterns among ceRNAs associated with NSCLC. WGCNA was used to identify the co-expression modules in NSCLC using the data from the TCGA and GTEx consortia. GTEx provides abundant data on gene expression from healthy people, thus benefitting the tumor purity quantification of the samples [41]. The WGCNA-brown mRNA module and the WGCNA-turquoise lncRNA module were the key modules for NSCLC. Among the predicted mRNAs, DEmRNAs, and mRNAs in the brown module, 1094 overlapping mRNAs were identified. Furthermore, a survival prognostic model with 13 genes (*FSTL3*, *CPS1*, *PTPN21*, *DEPDC1B*, *COL9A3*, *DSG2*, *LAMB1*, *STYK1*, *RBM6*, *DEPDC1*, *GTSE1*, *NAV3*, and *FKBP5*) was constructed for NSCLC through K–M estimator and Cox proportional hazard regression model.

Non-coding RNAs can promote *PD-L1*/*PD-1* expression through ceRNA regulation of NSCLC mechanisms [45]. However, given the complexity of the immune system, knowledge of a single biomarker that can precisely identify patients who can potentially benefit from ICIs is lacking. Immune-related gene signatures, immune infiltrating cells, and chemokines are important in predicting the clinical response to immunotherapy and prognosis of NSCLC patients [14]. A previous study shows that activated memory CD4 T cells and helper T cells are positively correlated with improvement in survival AMONG lung cancer patients [14]. By contrast, suppressive immune cells, including resting dendritic cells, M0 macrophages, and neutrophils, form an immunosuppressive microenvironment [46] and indicate a poorer prognosis. Integrated analysis of the correlation between ceRNA and TIME is necessary to improve the accuracy of prognostic predictions. The ceRNA signature in NSCLC is related to immune infiltrating cell types and PD-1 expression. Results from the execution of the CIBERSORT algorithm showed that the proportion of activated CD4 memory T cells and follicular helper T cells was positively correlated with the risk score of 13 gene-based ceRNA network, and the proportion of CD4 memory resting T cells, M0 macrophages, resting dendritic cells, and neutrophils was negatively associated with the 13 gene-based ceRNA risk score. Chemokines, especially *CXCL9*, *CXCL-10*, *and CXCL11/CXCR3* axis, play key roles in TIME and immune response [39]. Reduced levels of *CXCL9* and *CXCL10* are correlated with the immunosuppressive microenvironment, negative responses to ICIs, and poor prognosis among cancer patients [47]. In the present study, *CXCL9* and *CXCL10* were downregulated in the high-risk group, which further promoted immunosuppressive TIME. Taken together, our ceRNA risk model may effectively predict the TIME in NSCLC patients.

Furthermore, five mRNAs were obtained from the 13 gene-based prognostic model which corresponded to the three miRNAs selected from the prediction from among the 69 DElncRNAs. Based on these five mRNAs (*FKBP5*, *DEPDC1*, *CPS1*, *COL9A3*, and *PTPN21*), a ceRNA network that included 69 DElncRNAs and three DEmiRNAs (*miR-17-5p*, *miR-20b-5p*, and *miR-429*) was further established. Survival analysis revealed that seven DElncRNAs (*LINC00707*, *LINC00460*, *FEZF1-AS1*, *LINC00593*, *OTX2-AS1*, *LINC01833*, and *CASC8*) in the ceRNA network were associated with the OS in NSCLC patients. Finally, the prognostic ceRNAs from a coregulatory network involving five mRNAs from the 13-gene-based prognostic model and three DEmiRNAs were identified based on the seven-lncRNA signature.

Although chemotherapy and targeted anti-cancer therapies have improved the outcomes in NSCLC patients, resistance to chemotherapeutic and targeted drugs pose a serious challenge. The mechanisms of drug resistance remain unclear owing to the lack of approaches to accurately predict clinical responses to drugs. In this study, we predicted the association between agents and signatures in the ceRNA network using the pRRophetic algorithm. Our results demonstrated that the signatures in the ceRNA network were correlated with resistance to chemotherapeutic and targeted drugs, including cisplatin, paclitaxel, docetaxel, gemcitabine, and gefitinib, thus allowing prediction of drug response for developing personalized treatment of NSCLC patients.

In the five-gene signatures in the ceRNA network, the expression levels of *DEPDC1*, *CPS1*, *COL9A3*, and *PTPN21* were significantly different between early- and advanced-stage tumor tissues. Meanwhile, *FKBP5* was associated with a subtype of NSCLC. In progressive stages of NSCLC, *DEPDC1*, *CPS1*, and *COL9A3* were overexpressed, while *PTPN21* was down-regulated. *DEPDC1* is a putative oncogene [48] that can function as a transcriptional co-repressor during transcriptional regulation [49]. The results of a previous study strongly indicate that *DEPDC1* expression is positively related to the poor prognosis in NSCLC [50], which is consistent with the findings of the present study. Another previous study reports that *DEPDC1* promotes angiogenesis and invasion by activating chemokines in hepatocellular carcinoma [51]. *CPS1* is the mitochondrial enzyme involved in the first committed step of the urea cycle [52]. Its expression is positively correlated with tumor growth and is also associated with poor NSCLC prognosis [53]. *COL9A3* encodes instructions for generating one of the three alpha chains of type IX collagen [54] and is highly expressed in salivary adenoid cysts, breast basal-like carcinomas, and melanoma [55]. However, its function in cancer remains unknown. The correlation between *COL9A3* and NSCLC has not yet been reported. The present study is the first to show that abnormal *COL9A3* expression is associated with the tumor size in NSCLC patients. *PTPN21*, also called *PTPD-1*, is a member of the protein tyrosine phosphatase (PTP) family and controls cell growth, migration, and oncogenic transformation [56]. Given its inverse correlation with tumor invasion in bladder cancer tissues [57] and its immunosuppressive function [58], *PTPN21* may influence the early stages of tumor progression by inhibiting the immunosuppressive TIME and tumor invasion. This phenomenon could explain the high expression of *PTPN21* in the N0 stage.

*miR-17-5p*, *miR-20b-5p*, and *miR-429* are all involved in lung cancer [59–61]. *miR-429*, a member of the miRNA-200 family, functions as a tumor promoter in human NSCLC cells [62]. It is also positively correlated with the expression of immune checkpoints in NSCLC patients [63]. *miR-429* is known to regulate six lncRNAs (*LINC00707*, *LINC00460*, *FEZF1-AS1*, *LINC00593*, *LINC01833*, and *CASC8*) out of the seven lncRNAs in the proposed prognostic model. Among them, *LINC00707* is a novel long intergenic non-coding RNA that enhances sensitivity towards cisplatin in NSCLC cells [64], regulates mRNA stability, and can function as a potential diagnostic and prognostic marker in gastric cancer [65]. *LINC00460* promotes cell migration and invasion in NSCLC; it is a potential prognostic marker and a treatment target for patients with NSCLC [66]. *FEZF1-AS1* can promote the proliferation and invasion in tumor cells by suppressing Wnt/β-catenin signaling in NSCLC [67]. lncRNA *CASC8A* is a potential diagnostic biomarker for cancer [68] which can predict treatment response to platinum-based therapy and toxicity in patients with lung cancer [69]. Previous research findings corroborate our prediction of the ceRNA network and the prognostic models. Using the 13-mRNA-based prediction model and the seven-lncRNA-based prognostic model, we estimated the risk scores of NSCLC patients. The AUC of the ROC curve was > 0.65, which indicated that the prediction value had high accuracy in these prognostic models. Patients with NSCLC belonging to the high-risk group exhibited a high risk of progression according to the ceRNA network. Thus, these results provide a strong basis for the potential use of these novel predictive biomarkers for NSCLC prognosis and may guide molecular treatment approaches and further clinical research in NSCLC.

However, there were some inherent limitations in this study. First, was the lack of robust experimental verification. The function and mechanism of key ceRNAs should be further explored in the in vivo and in vitro settings. Second, was the need for prospective clinical trials to validate the prognosticators of the five-mRNA and seven-lncRNA signatures in the ceRNA network. Third, some superior computational algorithms could be used for biomarker identification [32–35] and prediction of miRNA-lncRNA interactions [36–38] in NSCLC. Finally, only the correlation between the 13-gene-based ceRNA prognostic model and NSCLC TIME was investigated, but further research on the predicted effect of the relevant lncRNAs in the microenvironment remains unknown. Despite these limitations, this study provided reliable prognostic markers that could be used to predict TIME and outcome in NSCLC patients. A multi-dimensional perspective was also proposed for the molecular regulatory mechanism of NSCLC through the TIME-related ceRNA network.

## Materials and methods

### TCGA and GTEx RNA sequencing datasets

The RNA-seq data for 455 LUAD samples and 489 LUSC samples were obtained from the TCGA dataset (https://gdc.cancer.gov/). All data for normal tissue samples were obtained from the GTEx Analysis V8 release version (https://gtexportal.org/home/datasets) which included a total of 578 lung samples. The miRNA sequence data of 455 tumor tissues with 45 control samples from LUAD patients and 489 tumor tissues with 45 adjacent non-cancerous lung tissues from LUSC patients were retrieved from the TCGA database.

### Identification of differentially expressed genes

The ensemble IDs of NSCLC samples were transformed into gene symbols using The Human GENCODE Gene Set-Release 34 version (https://www.gencodegenes.org/human/). The trimmed mean of the M-values was used to normalize the raw RNA-seq data. DEmRNAs, DElncRNAs, and DEmiRNAs between NSCLC and normal lung tissues were identified using the edgeR package in the Bioconductor project (version 3.11; http://www.bioconductor.org/). Absolute |logFC| > 1.5 and statistically adjusted *P* value of < 0.05 (*P* < 0.05) were considered as statistically significant DEmRNAs; |LogFC| > 1 and *P* < 0.05 were considered as statistically significant DEmiRNAs, and |LogFC| > 4 and *P* < 0.05 were considered as statistically significant DElncRNAs. The volcano map of DEmRNA and DEmiRNA expressions was visualized using the ggplot2 package (version 3.3.1, https://github.com/tidyverse/ggplot2) in R.

### Gene function and pathway enrichment analysis

ClusterProfiler R package [70] was used to perform GO functional annotation, KEGG pathway enrichment, and GSEA analyses for DEmRNAs. GO analysis was conducted to describe gene functions including BPs, cellular components (CCs), and molecular functions (MFs). KEGG-GSEA was performed for identification of the enriched signaling pathways, and *P* < 0.05 was considered as statistically significant enrichment.

### Co-expression network construction by WGCNA

The WGCNA package in R [71] was utilized to establish a co-expression network and identify the co-expression modules of mRNAs and lncRNAs. First, the variances of mRNAs and lncRNAs were computed by analyzing the variance across LUAD and LUSC samples. The top 80% mRNAs and the top 60% lncRNAs with the highest variances in WGCNA were chosen. Second, an appropriate soft-thresholding power (β) and a scale-free fit R^2 > 0.9 were set to establish a weighted adjacency matrix based on the scale-free topology criterion. After the modules with the highest correlation coefficient were analyzed, the adjacency matrix was transformed into a topological overlap matrix [72]. Co-expression modules were obtained using the dynamicTreeCut package in R, and their expression was analyzed by ME. After merging the highly correlated modules, the gene’s connectivity was calculated using the WGCNA package in R. Finally, GO-GSEA and KEGG analyses showed the functional enrichment of mRNAs in key modules.

### Prediction of target mRNAs

miRcode (http://www.mircode.org/) was used to predict interactions between the DEmiRNAs and the lncRNAs in the key modules that were most relevant to NSCLC. StarBase (http://starbase.sysu.edu.cn/), miRTarBase (http://mirtarbase.mbc.nctu.edu.tw/), miRDB (http://www.mirdb.org/), and TargetScan (http://www.targetscan.org/) databases were used to identify the target DEmRNAs from the predicted miRNAs.

### Construction of target mRNAs-related prognostic model

Clinical data of NSCLC patients were obtained to validate the potential prognostic applications of the target genes. The correlation between gene expression and patient survival was identified through univariate cox proportional hazard regression analysis using the “survival” package in R. Multivariate Cox proportional hazards regression model was subsequently utilized to construct the prognostic gene-based model. The risk score was calculated for each patient using the following formula:

$$Risk\;score={exp}_{mRNA1}\times \beta_{mRNA1}+\;{exp}_{mRNA2}\times \beta_{mRNA2}+\cdots +\;{exp}_{mRNAn}\times \beta_{mRNAn}$$

A high-risk score indicated a high risk of poor prognosis. The coefficient of genes, “β”, was calculated using the multivariate Cox regression model, and the expression of the corresponding mRNAs was represented as “exp” [73]. Patients with NSCLC were categorized into high- and low-risk groups with the medium value set as the demarcation point. Survival differences between the two groups were examined by K–M analysis and log-rank test. A ROC curve of risk score and the clinical features, including age, gender, and T, N, M, and TNM stages were plotted using the “survival ROC” package in R. The accuracy of the predictions of the prognostic model was evaluated using ROC curves. Associations between risk score or identified genes and clinical features were evaluated by stratified analysis. R software (version 3.6.1) was used for all statistical analyses.

### Analyses of tumor-infiltrating immune cells, PD-1, and chemokines

The fractions of 22 types of infiltrating immune cells, PD-1, and chemokines were identified using the gene expression data of NSCLC patients from the TCGA database to establish the relationship between ceRNA network and TIME. The differential immune infiltration levels were compared between the low- and high-risk groups using the CIBERSORT algorithm as previously described [74]. Only immune cells with a CIBERSORT *P* < 0.05 were selected for subsequent correlation analysis. The correlation coefficient between risk mRNAs in the ceRNA network and immune cell infiltration was evaluated through Pearson analysis.

### Construction of the ceRNA co-expression network

Competing endogenous RNA (ceRNA) networks were constructed using prognostic mRNA signatures, predicted miRNAs, and DElncRNAs. mRNA–miRNA interactions were identified using the miRDB, miRTarBase, StarBase, and TargetScan databases. The potential target lncRNAs for DEmiRNAs were identified using miRcode. Overlaps between mRNA–miRNA and lncRNA–miRNA interactions with prognostic mRNAs, DEmiRNAs, and DElncRNAs were further used to construct the ceRNA network. The open-source Cytoscape platform (https://cytoscape.org/) was used to visualize the regulatory relationships in the ceRNA network.

### Cox regression analyses for lncRNAs

A Cox proportional hazard regression analysis was performed for lncRNAs and mRNAs. Relevant associations between the lncRNAs in the ceRNA network and the OS in NSCLC patients were evaluated through the univariate Cox proportional hazard regression model. Prognostic lncRNAs were identified by the multivariate Cox regression model. The risk score formula for lncRNA was identical to that of mRNA and was as follows:

$$Risk\;score={exp}_{mRNA1}\times \beta_{mRNA1}+\;{exp}_{mRNA2}\times \beta_{mRNA2}+\ldots +\;{exp}_{mRNAn}\times \beta_{mRNAn}$$

A high-risk score indicated a high risk of poor prognosis. The regression coefficient of lncRNAs, “β”, was obtained through the multivariate Cox regression model. The expression of the corresponding lncRNAs was represented as “exp” [73]. ROC curve and clinicopathological correlation analyses were used to estimate the potential applicability of the prognostic signatures for predicting the NSCLC outcomes.

### Estimation of the sensitivity towards chemotherapeutic and targeted molecular agents

The IC50 values and sensitivity of chemotherapeutic and targeted therapeutic drugs were compared between the NSCLC subgroups classified by risk scores using the “pRRophetic” package in R. TCGA mRNA and lncRNA expression levels were analyzed based on the mRNA and lncRNA risk scores, respectively.

### Validation of the risk signatures from ceRNA network at protein level

The HPA database was used to validate the protein level expression of five risk mRNAs and compare them at the gene transcriptional level.

### Cell culture, RNA extraction, and RT-qPCR

The normal human bronchial epithelial cell line, BEAS-2B [75] was obtained from the State Key Laboratory of Oncology in South China. The cells were cultured in RPMI Medium 1640 (C11875500BT, Gibco, USA) supplemented with 10% fetal bovine serum (A31008-02, Gibco, USA) and 1% penicillin-streptomycin (PB180120, Procell Life Science & Technology Co. Ltd., China) in a 5% CO_2_ incubator at 37 °C. NSCLC cell lines, including PC-9 [76] (ZQ0487, Zhong Qiao Xin Zhou Biotechnology Co. Ltd., China), H1650 [77] (CL- 0166, Procell Life Science & Technology Co. Ltd., China), and H1975 [78] (CL-0298, Procell Life Science & Technology Co. Ltd., China), were cultured in Dulbecco’s Modified Eagle Medium (C11995500BT, Procell Life Science &Technology Co. Ltd., China) supplemented with 10% fetal bovine serum (A31008-02, Gibco, USA) and 1% penicillin-streptomycin (PB180120, Procell Life Science & Technology Co. Ltd., China) in a 5% CO_2_ incubator at 37 °C. Cells in the logarithmic growth phase were harvested for RNA extraction. Total RNA was isolated from the cells using the TRIzol reagent (15596026, Invitrogen, USA) and reverse transcribed into cDNA using the first-strand cDNA template through the PrimeScript RT reagent Kit with gDNA Eraser (RR047A, TaKaRa, Japan). SYBR Premix Ex Taq II (RR820A, TaKaRa, Japan) was used for amplification on the CXF96 System (BioRad, United States). *ACTB* served as the endogenous control. The primer designs are listed in S1 Table. lncRNA expression was normalized to that of *ACTB* and calculated using the 2^−ΔΔCq^ method.

### Statistical analysis

Statistical analyses for bioinformatic experiments were performed using the previously mentioned R packages in the R software (v.3.6.3). Differential expression levels between groups through RT-qPCR analyses were compared using the parametric one-way ANOVA in GraphPad Prism version 8.0 (GraphPad Software) and SPSS 23.0 software (IBM). Statistical significance was considered at *P* < 0.05.

## Supporting information

S1 Table Real-time quantitative PCR primer sequences used in this study. (DOCX)

## Abbreviations

AUC: the area under the curve
BP: biological process
CC: cellular component
ceRNAs: Competing endogenous RNAs
CIBERSORT: Estimating Relative Subsets of RNA Transcripts
DElncRNAs: differentially expressed lncRNAs
DEmiRNAs: differentially expressed miRNAs
DEmRNAs: differentially expressed mRNAs
edgeR: empirical analysis of digital gene expression in R
GO: Gene Ontology
GSEA: Gene Set Enrichment Analysis
GTEx: the Genotype-Tissue Expression database
HPA database: The Human Protein Atlas database
IC50: half-maximal inhibitory concentration
ICI: Immune checkpoints inhibitor
KEGG: Kyoto Encyclopedia of Genes and Genomes pathway analyses
K-M: Kaplan–Meier survival analysis
lncRNA: long non-coding RNA
logFC: log (fold change)
LUAD: Lung adenocarcinoma
LUSC: lung squamous cell carcinoma
ME: module eigengene
MF: molecular function
miRNA: microRNA
MRE: miRNA response element
NSCLC: non-small cell lung cancer
OS: overall survival
PD-1: programmed cell death-1
*PD-L1*: programmed cell death ligand-1
ROC: receiver operating characteristic curve analysis
RT-qPCR: reverse transcription quantitative polymerase chain reaction
TCGA: the Cancer Genome Atlas database
TIME: tumor immune microenvironment
TKI: tyrosine kinase inhibitor
TOM: topological overlap matrix
WGCNA: weighted gene co-expression network analysis

## References

[1] Siegel RL, Miller KD, Jemal A. Cancer statistics, 2019. CA Cancer J. Clin. 2019;69(1):7–34. Epub 2019/01/09. doi: 10.3322/caac.21551 . 30620402

[2] Miller KD, Nogueira L, Mariotto AB, Rowland JH, Yabroff KR, Alfano CM, et al. Cancer treatment and survivorship statistics, 2019. CA Cancer J. Clin. 2019;69(5):363–385. Epub 2019/06/12. doi: 10.3322/caac.21565 . 31184787

[3] Chen Z, Fillmore CM, Hammerman PS, Kim CF, Wong KK. Non-small-cell lung cancers: a heterogeneous set of diseases. Nat Rev Cancer. 2014;14(8):535–546. Epub 2014/07/25. doi: 10.1038/nrc3775 . 25056707 PMC5712844

[4] Goldstraw P, Chansky K, Crowley J, Rami-Porta R, Asamura H, Eberhardt WE, et al. The IASLC Lung Cancer Staging Project: Proposals for Revision of the TNM Stage Groupings in the Forthcoming (Eighth) Edition of the TNM Classification for Lung Cancer. J. Thorac. Oncol. 2016;11(1):39–51. Epub 2016/01/15. doi: 10.1016/j.jtho.2015.09.009 . 26762738

[5] Howlader NNA, Krapcho M, Miller D, Brest A, Yu M, Ruhl J, (eds). SEER Cancer Statistics Review, 1975–2017, National Cancer Institute. Bethesda, MD. 2020 [updated April 2020]. https://seer.cancer.gov/csr/1975_2017/.

[6] Liang W, Zhao Y, Huang W, Gao Y, Xu W, Tao J, et al. Non-invasive diagnosis of early-stage lung cancer using high-throughput targeted DNA methylation sequencing of circulating tumor DNA (ctDNA). Theranostics. 2019;9(7):2056–2070. Epub 2019/05/01. doi: 10.7150/thno.28119 . 31037156 PMC6485294

[7] de Koning HJ, Meza R, Plevritis SK, ten Haaf K, Munshi VN, Jeon J, et al. Benefits and harms of computed tomography lung cancer screening strategies: a comparative modeling study for the U.S. Preventive Services Task Force. Ann. Intern. Med. 2014;160(5):311–320. Epub 2014/01/01. doi: 10.7326/M13-2316 . 24379002 PMC4116741

[8] Vargas AJ, Harris CC. Biomarker development in the precision medicine era: lung cancer as a case study. Nature reviews. Cancer. 2016;16(8):525–537. Epub 2016/07/08. . 27388699 10.1038/nrc.2016.56 PMC6662593

[9] Lamberti G, Andrini E, Sisi M, Rizzo A, Parisi C, Di Federico A, et al. Beyond EGFR, ALK and ROS1: Current evidence and future perspectives on newly targetable oncogenic drivers in lung adenocarcinoma. Crit Rev Oncol Hematol. 2020;156:103119. Epub 2020/10/15. doi: 10.1016/j.critrevonc.2020.103119 . 33053439

[10] Rotow J, Bivona TG. Understanding and targeting resistance mechanisms in NSCLC. Nat Rev Cancer. 2017;17(11):637–658. Epub 2017/10/27. doi: 10.1038/nrc.2017.84 . 29068003

[11] Paz-Ares L, Luft A, Vicente D, Tafreshi A, Gümüş M, Mazières J, et al. Pembrolizumab plus Chemotherapy for Squamous Non-Small-Cell Lung Cancer. New Engl. J. Med. 2018;379(21):2040–2051. Epub 2018/10/04. doi: 10.1056/NEJMoa1810865 . 30280635

[12] Pathak R, Pharaon RR, Mohanty A, Villaflor VM, Salgia R, Massarelli E. Acquired Resistance to PD-1/PD-L1 Blockade in Lung Cancer: Mechanisms and Patterns of Failure. Cancers (Basel). 2020;12(12). Epub 2021/01/10. doi: 10.3390/cancers12123851 . 33419311 PMC7767234

[13] Sequist LV, Han JY, Ahn MJ, Cho BC, Yu H, Kim SW, et al. Osimertinib plus savolitinib in patients with EGFR mutation-positive, MET-amplified, non-small-cell lung cancer after progression on EGFR tyrosine kinase inhibitors: interim results from a multicentre, open-label, phase 1b study. Lancet Oncol. 2020;21(3):373–386. Epub 2020/02/07. doi: 10.1016/S1470-2045(19)30785-5 . 32027846

[14] Catacchio I, Scattone A, Silvestris N, Mangia A. Immune Prophets of Lung Cancer: The Prognostic and Predictive Landscape of Cellular and Molecular Immune Markers. Transl. Oncol. 2018;11(3):825–835. Epub 2018/05/08. doi: 10.1016/j.tranon.2018.04.006 . 29729581 PMC6050352

[15] Brody R, Zhang Y, Ballas M, Siddiqui MK, Gupta P, Barker C, et al. PD-L1 expression in advanced NSCLC: Insights into risk stratification and treatment selection from a systematic literature review. Lung Cancer. 2017;112:200–215. Epub 2017/12/02. doi: 10.1016/j.lungcan.2017.08.005 . 29191596

[16] Rivas-Fuentes S, Salgado-Aguayo A, Pertuz Belloso S, Gorocica Rosete P, Alvarado-Vásquez N, Aquino-Jarquin G. Role of Chemokines in Non-Small Cell Lung Cancer: Angiogenesis and Inflammation. J. Cancer. 2015;6(10):938–952. Epub 2015/09/01. doi: 10.7150/jca.12286 . 26316890 PMC4543754

[17] Salmena L, Poliseno L, Tay Y, Kats L, Pandolfi PP. A ceRNA hypothesis: the Rosetta Stone of a hidden RNA language? Cell. 2011;146(3):353–358. Epub 2011/08/02. doi: 10.1016/j.cell.2011.07.014 . 21802130 PMC3235919

[18] Wang H, Huo X, Yang XR, He J, Cheng L, Wang N, et al. STAT3-mediated upregulation of lncRNA HOXD-AS1 as a ceRNA facilitates liver cancer metastasis by regulating SOX4. Mol. Cancer. 2017;16(1):136. Epub 2017/08/16. doi: 10.1186/s12943-017-0680-1 . 28810927 PMC5558651

[19] Yang J, Qiu Q, Qian X, Yi J, Jiao Y, Yu M, et al. Long noncoding RNA LCAT1 functions as a ceRNA to regulate RAC1 function by sponging miR-4715-5p in lung cancer. Mol. Cancer. 2019;18(1):171. Epub 2019/11/30. doi: 10.1186/s12943-019-1107-y . 31779616 PMC6883523

[20] Chen L, Nan A, Zhang N, Jia Y, Li X, Ling Y, et al. Circular RNA 100146 functions as an oncogene through direct binding to miR-361-3p and miR-615-5p in non-small cell lung cancer. Mol. Cancer. 2019;18(1):13. Epub 2019/01/23. doi: 10.1186/s12943-019-0943-0 . 30665425 PMC6340182

[21] Jiang J, Bi Y, Liu X-P, Yu D, Yan X, Yao J, et al. To construct a ceRNA regulatory network as prognostic biomarkers for bladder cancer. J. Cell. Mol. Med. 2020;24(9):5375–5386. Epub 2020/03/31. doi: 10.1111/jcmm.15193 . 32233022 PMC7205833

[22] Liu N, Liu Z, Liu X, Chen H. Comprehensive Analysis of a Competing Endogenous RNA Network Identifies Seven-lncRNA Signature as a Prognostic Biomarker for Melanoma. Frontiers in oncology. 2019;9:935–935. doi: 10.3389/fonc.2019.00935 . 31649871 PMC6794712

[23] Wang XW, Guo QQ, Wei Y, Ren KM, Zheng FS, Tang J, et al. Construction of a competing endogenous RNA network using differentially expressed lncRNAs, miRNAs and mRNAs in non-small cell lung cancer. Oncol. Rep. 2019;42(6):2402–2415. Epub 2019/10/23. doi: 10.3892/or.2019.7378 . 31638248 PMC6859443

[24] Li S, Cui Z, Zhao Y, Ma S, Sun Y, Li H, et al. Candidate lncRNA-microRNA-mRNA networks in predicting non-small cell lung cancer and related prognosis analysis. J. Cancer Res. Clin. Oncol. 2020;146(4):883–896. Epub 2020/03/04. doi: 10.1007/s00432-020-03161-6 . 32124023 PMC11804401

[25] Li Y, Sun N, Lu Z, Sun S, Huang J, Chen Z, et al. Prognostic alternative mRNA splicing signature in non-small cell lung cancer. Cancer Lett. 2017;393:40–51. Epub 2017/02/23. doi: 10.1016/j.canlet.2017.02.016 . 28223168

[26] Aran D, Camarda R, Odegaard J, Paik H, Oskotsky B, Krings G, et al. Comprehensive analysis of normal adjacent to tumor transcriptomes. Nat Commun. 2017;8(1):1077. Epub 2017/10/24. doi: 10.1038/s41467-017-01027-z . 29057876 PMC5651823

[27] Consortium G. The Genotype-Tissue Expression (GTEx) project. Nat. Genet. 2013;45(6):580–585. Epub 2013/05/30. doi: 10.1038/ng.2653 . 23715323 PMC4010069

[28] Unfried JP, Serrano G, Suárez B, Sangro P, Ferretti V, Prior C, et al. Identification of Coding and Long Noncoding RNAs Differentially Expressed in Tumors and Preferentially Expressed in Healthy Tissues. Cancer Res. 2019;79(20):5167–5180. Epub 2019/08/08. doi: 10.1158/0008-5472.CAN-19-0400 . 31387921

[29] Li N, Zhan X. Identification of clinical trait-related lncRNA and mRNA biomarkers with weighted gene co-expression network analysis as useful tool for personalized medicine in ovarian cancer. The EPMA journal. 2019;10(3):273–290. Epub 2019/08/30. doi: 10.1007/s13167-019-00175-0 . 31462944 PMC6695468

[30] Seung SJ, Hurry M, Walton RN, Evans WK. Real-world treatment patterns and survival in stage IV non-small-cell lung cancer in Canada. Current oncology (Toronto, Ont.). 2020;27(4):e361–e367. Epub 2020/09/10. doi: 10.3747/co.27.6049 . 32905294 PMC7467785

[31] Gray JE, Villegas A, Daniel D, Vicente D, Murakami S, Hui R, et al. Three-Year Overall Survival with Durvalumab after Chemoradiotherapy in Stage III NSCLC-Update from PACIFIC. J. Thorac. Oncol. 2020;15(2):288–293. Epub 2019/10/18. doi: 10.1016/j.jtho.2019.10.002 . 31622733 PMC7244187

[32] Chen X, Wang L, Qu J, Guan NN, Li JQ. Predicting miRNA-disease association based on inductive matrix completion. Bioinformatics. 2018;34(24):4256–4265. Epub 2018/06/26. doi: 10.1093/bioinformatics/bty503 . 29939227

[33] Chen X, Xie D, Zhao Q, You ZH. MicroRNAs and complex diseases: from experimental results to computational models. Brief Bioinform. 2019;20(2):515–539. Epub 2017/10/19. doi: 10.1093/bib/bbx130 . 29045685

[34] Chen X, Yan CC, Zhang X, You ZH. Long non-coding RNAs and complex diseases: from experimental results to computational models. Brief Bioinform. 2017;18(4):558–576. Epub 2016/06/28. doi: 10.1093/bib/bbw060 . 27345524 PMC5862301

[35] Chen X, Zhu CC, Yin J. Ensemble of decision tree reveals potential miRNA-disease associations. PLoS Comp. Biol. 2019;15(7):e1007209. Epub 2019/07/23. doi: 10.1371/journal.pcbi.1007209 . 31329575 PMC6675125

[36] Liu H, Ren G, Chen H, Liu Q, Yang Y, Zhao Q. Predicting lncRNA–miRNA interactions based on logistic matrix factorization with neighborhood regularized. Knowl-Based Syst. 2020;191:105261. doi: 10.1016/j.knosys.2019.105261

[37] Zhang L, Liu T, Chen H, Zhao Q, Liu H. Predicting lncRNA-miRNA interactions based on interactome network and graphlet interaction. Genomics. 2021;113(3):874–880. Epub 2021/02/16. doi: 10.1016/j.ygeno.2021.02.002 . 33588070

[38] Zhang L, Yang P, Feng H, Zhao Q, Liu H. Using Network Distance Analysis to Predict lncRNA-miRNA Interactions. Interdisciplinary sciences, computational life sciences. 2021;13(3):535–545. Epub 2021/07/08. doi: 10.1007/s12539-021-00458-z . 34232474

[39] Tokunaga R, Zhang W, Naseem M, Puccini A, Berger MD, Soni S, et al. CXCL9, CXCL10, CXCL11/CXCR3 axis for immune activation—A target for novel cancer therapy. Cancer Treat. Rev. 2018;63:40–47. Epub 2017/11/26. doi: 10.1016/j.ctrv.2017.11.007 . 29207310 PMC5801162

[40] Wang X, Yin H, Zhang L, Zheng D, Yang Y, Zhang J, et al. The construction and analysis of the aberrant lncRNA-miRNA-mRNA network in non-small cell lung cancer. J. Thorac. Dis. 2019;11(5):1772–1778. doi: 10.21037/jtd.2019.05.69 . 31285869 PMC6588744

[41] Mounir M, Lucchetta M, Silva TC, Olsen C, Bontempi G, Chen X, et al. New functionalities in the TCGAbiolinks package for the study and integration of cancer data from GDC and GTEx. PLoS Comp. Biol. 2019;15(3):e1006701. Epub 2019/03/06. doi: 10.1371/journal.pcbi.1006701 . 30835723 PMC6420023

[42] Downing JR, Wilson RK, Zhang J, Mardis ER, Pui C-H, Ding L, et al. The Pediatric Cancer Genome Project. Nat. Genet. 2012;44(6):619–622. doi: 10.1038/ng.2287 . 22641210 PMC3619412

[43] Frazee AC, Langmead B, Leek JT. ReCount: a multi-experiment resource of analysis-ready RNA-seq gene count datasets. BMC Bioinformatics. 2011;12:449. Epub 2011/11/18. doi: 10.1186/1471-2105-12-449 . 22087737 PMC3229291

[44] Knudsen ES, Kumarasamy V, Ruiz A, Sivinski J, Chung S, Grant A, et al. Cell cycle plasticity driven by MTOR signaling: integral resistance to CDK4/6 inhibition in patient-derived models of pancreatic cancer. Oncogene. 2019;38(18):3355–3370. Epub 2019/01/31. doi: 10.1038/s41388-018-0650-0 . 30696953 PMC6499706

[45] Wang J, Zhao X, Wang Y, Ren F, Sun D, Yan Y, et al. circRNA-002178 act as a ceRNA to promote PDL1/PD1 expression in lung adenocarcinoma. Cell Death Dis. 2020;11(1):32. Epub 2020/01/18. doi: 10.1038/s41419-020-2230-9 . 31949130 PMC6965119

[46] Nurieva R, Wang J, Sahoo A. T-cell tolerance in cancer. Immunotherapy. 2013;5(5):513–531. doi: 10.2217/imt.13.33 . 23638746 PMC5103631

[47] Nagarsheth N, Wicha MS, Zou W. Chemokines in the cancer microenvironment and their relevance in cancer immunotherapy. Nature reviews. Immunology. 2017;17(9):559–572. Epub 2017/05/30. doi: 10.1038/nri.2017.49 . 28555670 PMC5731833

[48] Kanehira M, Harada Y, Takata R, Shuin T, Miki T, Fujioka T, et al. Involvement of upregulation of DEPDC1 (DEP domain containing 1) in bladder carcinogenesis. Oncogene. 2007;26(44):6448–6455. Epub 2007/04/25. doi: 10.1038/sj.onc.1210466 . 17452976

[49] Harada Y, Kanehira M, Fujisawa Y, Takata R, Shuin T, Miki T, et al. Cell-permeable peptide DEPDC1-ZNF224 interferes with transcriptional repression and oncogenicity in bladder cancer cells. Cancer Res. 2010;70(14):5829–5839. Epub 2010/07/01. doi: 10.1158/0008-5472.CAN-10-0255 . 20587513

[50] Okayama H, Kohno T, Ishii Y, Shimada Y, Shiraishi K, Iwakawa R, et al. Identification of genes upregulated in ALK-positive and EGFR/KRAS/ALK-negative lung adenocarcinomas. Cancer Res. 2012;72(1):100–111. Epub 2011/11/15. doi: 10.1158/0008-5472.CAN-11-1403 . 22080568

[51] Guo W, Li H, Liu H, Ma X, Yang S, Wang Z. DEPDC1 drives hepatocellular carcinoma cell proliferation, invasion and angiogenesis by regulating the CCL20/CCR6 signaling pathway. Oncol. Rep. 2019;42(3):1075–1089. Epub 2019/07/05. doi: 10.3892/or.2019.7221 . 31322256 PMC6667871

[52] Çeliktas M, Tanaka I, Tripathi SC, Fahrmann JF, Aguilar-Bonavides C, Villalobos P, et al. Role of CPS1 in Cell Growth, Metabolism and Prognosis in LKB1-Inactivated Lung Adenocarcinoma. J. Natl. Cancer Inst. 2017;109(3):1–9. Epub 2017/04/05. doi: 10.1093/jnci/djw231 . 28376202 PMC5198847

[53] Kim J, Hu Z, Cai L, Li K, Choi E, Faubert B, et al. CPS1 maintains pyrimidine pools and DNA synthesis in KRAS/LKB1-mutant lung cancer cells. Nature. 2017;546(7656):168–172. Epub 2017/05/26. doi: 10.1038/nature22359 . 28538732 PMC5472349

[54] Paassilta P, Lohiniva J, Annunen S, Bonaventure J, Le Merrer M, Pai L, et al. COL9A3: A third locus for multiple epiphyseal dysplasia. Am. J. Hum. Genet. 1999;64(4):1036–1044. Epub 1999/03/26. doi: 10.1086/302328 . 10090888 PMC1377827

[55] Ivanov SV, Panaccione A, Nonaka D, Prasad ML, Boyd KL, Brown B, et al. Diagnostic SOX10 gene signatures in salivary adenoid cystic and breast basal-like carcinomas. Br. J. Cancer. 2013;109(2):444–451. Epub 2013/06/27. doi: 10.1038/bjc.2013.326 . 23799842 PMC3721393

[56] Alonso A, Sasin J, Bottini N, Friedberg I, Friedberg I, Osterman A, et al. Protein tyrosine phosphatases in the human genome. Cell. 2004;117(6):699–711. Epub 2004/06/10. doi: 10.1016/j.cell.2004.05.018 . 15186772

[57] Carlucci A, Porpora M, Garbi C, Galgani M, Santoriello M, Mascolo M, et al. PTPD1 supports receptor stability and mitogenic signaling in bladder cancer cells. J. Biol. Chem. 2010;285(50):39260–39270. Epub 2010/10/07. doi: 10.1074/jbc.M110.174706 . 20923765 PMC2998146

[58] Wang H, Ye X, Xiao H, Zhu N, Wei C, Sun X, et al. PTPN21 Overexpression Promotes Osteogenic and Adipogenic Differentiation of Bone Marrow-Derived Mesenchymal Stem Cells but Inhibits the Immunosuppressive Function. Stem Cells Int. 2019;2019:4686132. Epub 2019/12/31. doi: 10.1155/2019/4686132 . 31885609 PMC6907062

[59] Li Y, Liang M, Zhang Y, Yuan B, Gao W, Shi Z, et al. miR-93, miR-373, and miR-17-5p Negatively Regulate the Expression of TBP2 in Lung Cancer. Front Oncol. 2020;10:526. Epub 2020/05/20. doi: 10.3389/fonc.2020.00526 . 32426273 PMC7212423

[60] Li C, Qin F, Hu F, Xu H, Sun G, Han G, et al. Characterization and selective incorporation of small non-coding RNAs in non-small cell lung cancer extracellular vesicles. Cell & bioscience. 2018;8:2. Epub 2018/01/19. doi: 10.1186/s13578-018-0202-x . 29344346 PMC5763536

[61] Lang Y, Xu S, Ma J, Wu J, Jin S, Cao S, et al. MicroRNA-429 induces tumorigenesis of human non-small cell lung cancer cells and targets multiple tumor suppressor genes. Biochem. Biophys. Res. Commun. 2014;450(1):154–159. Epub 2014/05/29. doi: 10.1016/j.bbrc.2014.05.084 . 24866238

[62] Liu C, Hu W, Li LL, Wang YX, Zhou Q, Zhang F, et al. Roles of miR-200 family members in lung cancer: more than tumor suppressors. Future oncology (London, England). 2018;14(27):2875–2886. Epub 2018/09/14. doi: 10.2217/fon-2018-0155 . 30208739

[63] Grenda A, Nicoś M, Szczyrek M, Krawczyk P, Kucharczyk T, Jarosz B, et al. MicroRNAs aid the assessment of programmed death ligand 1 expression in patients with non-small cell lung cancer. Oncol. Lett. 2019;17(6):5193–5200. Epub 2019/06/13. doi: 10.3892/ol.2019.10207 . 31186735 PMC6507482

[64] Zhang H, Luo Y, Xu W, Li K, Liao C. Silencing long intergenic non-coding RNA 00707 enhances cisplatin sensitivity in cisplatin-resistant non-small-cell lung cancer cells by sponging miR-145. Oncol. Lett. 2019;18(6):6261–6268. Epub 2019/12/04. doi: 10.3892/ol.2019.10959 . 31788103 PMC6865129

[65] Xie M, Ma T, Xue J, Ma H, Sun M, Zhang Z, et al. The long intergenic non-protein coding RNA 707 promotes proliferation and metastasis of gastric cancer by interacting with mRNA stabilizing protein HuR. Cancer Lett. 2019;443:67–79. Epub 2018/12/07. doi: 10.1016/j.canlet.2018.11.032 . 30502359

[66] Li K, Sun D, Gou Q, Ke X, Gong Y, Zuo Y, et al. Long non-coding RNA linc00460 promotes epithelial-mesenchymal transition and cell migration in lung cancer cells. Cancer Lett. 2018;420:80–90. Epub 2018/02/08. doi: 10.1016/j.canlet.2018.01.060 . 29409808

[67] He R, Zhang FH, Shen N. LncRNA FEZF1-AS1 enhances epithelial-mesenchymal transition (EMT) through suppressing E-cadherin and regulating WNT pathway in non-small cell lung cancer (NSCLC). Biomedicine & pharmacotherapy = Biomedecine & pharmacotherapie. 2017;95:331–338. Epub 2017/09/01. doi: 10.1016/j.biopha.2017.08.057 . 28858731

[68] Cui Z, Gao M, Yin Z, Yan L, Cui L. Association between lncRNA CASC8 polymorphisms and the risk of cancer: a meta-analysis. Cancer Manag. Res. 2018;10:3141–3148. Epub 2018/09/15. doi: 10.2147/CMAR.S170783 . 30214306 PMC6124472

[69] Hu L, Chen SH, Lv QL, Sun B, Qu Q, Qin CZ, et al. Clinical Significance of Long Non-Coding RNA CASC8 rs10505477 Polymorphism in Lung Cancer Susceptibility, Platinum-Based Chemotherapy Response, and Toxicity. Int. J. Env. Res. Public Health. 2016;13(6). Epub 2016/06/02. doi: 10.3390/ijerph13060545 . 27249003 PMC4924002

[70] Yu G, Wang L-G, Han Y, He Q-Y. clusterProfiler: an R Package for Comparing Biological Themes Among Gene Clusters. OMICS: A Journal of Integrative Biology. 2012;16(5):284–287. doi: 10.1089/omi.2011.0118 22455463 PMC3339379

[71] Langfelder P, Horvath S. WGCNA: an R package for weighted correlation network analysis. BMC Bioinformatics. 2008;9:559. Epub 2008/12/31. doi: 10.1186/1471-2105-9-559 . 19114008 PMC2631488

[72] Li A, Horvath S. Network module detection: Affinity search technique with the multi-node topological overlap measure. BMC research notes. 2009;2:142. Epub 2009/07/22. doi: 10.1186/1756-0500-2-142 . 19619323 PMC2727520

[73] Zeng JH, Liang L, He RQ, Tang RX, Cai XY, Chen JQ, et al. Comprehensive investigation of a novel differentially expressed lncRNA expression profile signature to assess the survival of patients with colorectal adenocarcinoma. Oncotarget. 2017;8(10):16811–16828. Epub 2017/02/12. doi: 10.18632/oncotarget.15161 . 28187432 PMC5370003

[74] Newman AM, Liu CL, Green MR, Gentles AJ, Feng W, Xu Y, et al. Robust enumeration of cell subsets from tissue expression profiles. Nat. Methods. 2015;12(5):453–457. Epub 2015/03/31. doi: 10.1038/nmeth.3337 . 25822800 PMC4739640

[75] Amstad P, Reddel RR, Pfeifer A, Malan-Shibley L, Mark GE 3rd, Harris CC. Neoplastic transformation of a human bronchial epithelial cell line by a recombinant retrovirus encoding viral Harvey ras. Mol. Carcinog. 1988;1(3):151–160. Epub 1988/01/01. doi: 10.1002/mc.2940010303 . 2855021

[76] Lee YC, Saijo N, Sasaki Y, Takahashi H, Sakurai M, Ishihara J, et al. Clonogenic patterns of human pulmonary adenocarcinoma cell lines (PC-9, PC-13 and PC-14) and how they influence the results of test for chemosensitivity to cisplatin in the human tumor clonogenic assay. Jap. J. Clin. Oncol. 1985;15(4):637–644. Epub 1985/12/01. 4094096

[77] Zhang YA, Nemunaitis J, Scanlon KJ, Tong AW. Anti-tumorigenic effect of a K-ras ribozyme against human lung cancer cell line heterotransplants in nude mice. Gene Ther. 2000;7(23):2041–2050. Epub 2001/02/15. doi: 10.1038/sj.gt.3301331 . 11175317

[78] Mambo E, Chatterjee A, de Souza-Pinto NC, Mayard S, Hogue BA, Hoque MO, et al. Oxidized guanine lesions and hOgg1 activity in lung cancer. Oncogene. 2005;24(28):4496–4508. Epub 2005/04/28. doi: 10.1038/sj.onc.1208669 . 15856018

