# Supplementary material for: Comprehensive analysis of an immune infiltrate-related competitive endogenous RNA network reveals potential prognostic biomarkers for non-small cell lung cancer
Source: PLoS One. 2021 Dec 2;16(12):e0260720. doi: 10.1371/journal.pone.0260720 (PMC8639052; doi:10.1371/journal.pone.0260720)
Supplement: S1 Table — (DOCX) [file pone.0260720.s001.docx]

**S1 Table. Real‐time quantitative PCR primer sequences used in this study**

| Primer name | Forward (5′‐3′) | Reverse (5′‐3′) |
| --- | --- | --- |
| ACTB | CAGGGCGTGATGGTGGGCA | CAAACATCATCTGGGTCATCTTCTC |
| LINC01833 | GCCTCGCTCTCTCATCTGAAAGTG | TGGAGGGTGGTCATCCGACTTG |
| OTX2-AS1 | CTCTCTCCCTCTCTCCCTCTCTCC | AGTGAGCAGTGATGGTGCCAATG |
| LINC00593 | CCAAGCAATTAGCCTCCAGGGAAG | GAGATCAAGGTGTCAGCAGGGTTG |
| LINC00460 | TCGGCTAAGAGTCACCCTGGATG | CACAGACGCCTCCCACACAATG |
| LINC00707 | TCAAATCCCGCCTCTACTCCATCC | GTCCAGGTAAGCTCTGTGTCATCG |
| CASC8 | TGCCTCACCTGCCTCACCAC | ATGCCAGGGCTACAGCAATCAAC |
